# LnCeCell 2.0: an updated resource for lncRNA-associated ceRNA networks and web tools based on single-cell and spatial transcriptomics sequencing data

**DOI:** 10.1093/nar/gkae947

**Published:** 2024-10-29

**Authors:** Qiuyan Guo, Qian Liu, Danni He, Mengyu Xin, Yifan Dai, Rui Sun, Houxing Li, Yujie Zhang, Jiatong Li, Congcong Kong, Yue Gao, Hui Zhi, Feng Li, Shangwei Ning, Peng Wang

**Affiliations:** Department of Gynecology, the First Affiliated Hospital of Harbin Medical University, 23 Youzheng Road, Harbin 150081, China; College of Bioinformatics Science and Technology, Harbin Medical University, 157 Baojian Road, Harbin 150081, China; College of Bioinformatics Science and Technology, Harbin Medical University, 157 Baojian Road, Harbin 150081, China; College of Bioinformatics Science and Technology, Harbin Medical University, 157 Baojian Road, Harbin 150081, China; College of Bioinformatics Science and Technology, Harbin Medical University, 157 Baojian Road, Harbin 150081, China; College of Bioinformatics Science and Technology, Harbin Medical University, 157 Baojian Road, Harbin 150081, China; College of Bioinformatics Science and Technology, Harbin Medical University, 157 Baojian Road, Harbin 150081, China; College of Bioinformatics Science and Technology, Harbin Medical University, 157 Baojian Road, Harbin 150081, China; College of Bioinformatics Science and Technology, Harbin Medical University, 157 Baojian Road, Harbin 150081, China; Department of Gynecology, the First Affiliated Hospital of Harbin Medical University, 23 Youzheng Road, Harbin 150081, China; College of Bioinformatics Science and Technology, Harbin Medical University, 157 Baojian Road, Harbin 150081, China; College of Bioinformatics Science and Technology, Harbin Medical University, 157 Baojian Road, Harbin 150081, China; College of Bioinformatics Science and Technology, Harbin Medical University, 157 Baojian Road, Harbin 150081, China; College of Bioinformatics Science and Technology, Harbin Medical University, 157 Baojian Road, Harbin 150081, China; College of Bioinformatics Science and Technology, Harbin Medical University, 157 Baojian Road, Harbin 150081, China

## Abstract

We describe LnCeCell 2.0 (http://bio-bigdata.hrbmu.edu.cn/LnCeCell), an updated resource for lncRNA-associated competing endogenous RNA (ceRNA) networks and web tools based on single-cell and spatial transcriptomics sequencing (stRNA-seq) data. We have updated the LnCeCell 2.0 database with significantly expanded data and improved features, including (i) 257 single-cell RNA sequencing and stRNA-seq datasets across 86 diseases/phenotypes and 80 human normal tissues, (ii) 836 581 cell-specific and spatial spot-specific ceRNA interactions and functional networks for 1 002 988 cells and 367 971 spatial spots, (iii) 15 489 experimentally supported lncRNA biomarkers related to disease pathology, diagnosis and treatment, (iv) detailed annotation of cell type, cell state, subcellular and extracellular locations of ceRNAs through manual curation and (v) ceRNA expression profiles and follow-up clinical information of 20 326 cancer patients. Further, a panel of 24 flexible tools (including 8 comprehensive and 16 mini-analysis tools) was developed to investigate ceRNA-regulated mechanisms at single-cell/spot resolution. The *CeCellTraject* tool, for example, illustrates the detailed ceRNA distribution of different cell populations and explores the dynamic change of the ceRNA network along the developmental trajectory. LnCeCell 2.0 will facilitate the study of fine-tuned lncRNA-ceRNA networks with single-cell and spatial spot resolution, helping us to understand the regulatory mechanisms behind complex microbial ecosystems.

## Graphical Abstract

**Graphical Abstract:**
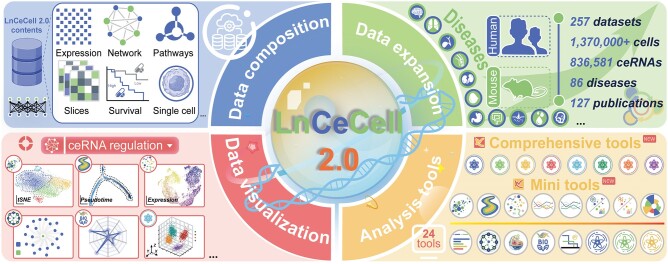

## Introduction

Long non-coding RNAs (lncRNAs) are increasingly recognized as critical regulatory molecules in diverse disease states, including cancers. LncRNAs can modulate chromatin function, regulate the assembly and function of membraneless nucleosomes, alter cytoplasmic messenger RNA (mRNA) stability and translation, and interfere with signalling pathways, many of which ultimately affect gene expression in diverse biological and physiopathological contexts (1–3). In particular, with regard to the development and metastasis of many cancers, lncRNAs have been identified as potential oncogenic biomarkers (4). It is evident that the regulatory mechanisms of biological processes mediated by lncRNAs frequently involve microRNAs (miRNAs) through a competing endogenous RNA (ceRNA) theory, thereby forming an lncRNA–miRNA–mRNA axis to co-regulate gene expression (5,6). In other words, lncRNAs could act as ‘miRNA sponges’ by competing for binding to shared miRNAs and indirectly regulating mRNA expression. These lncRNA-associated ceRNAs are widely studied and significantly expanding the functional genetic information in the human genome (6). For example, the LINC00680/miR-423-5p/PAK6 axis could serve as a prognostic biomarker and therapeutic target for oesophageal squamous cell carcinoma (7). Multiple ceRNA axes may co-operate to regulate cancer progression through common lncRNAs, miRNAs or mRNAs that form a ceRNA network (8). For example, lncRNA BC069792 acted as a molecular sponge, adsorbing has-miR-658 and has-miR-4739 to upregulate KCNQ4 protein expression, inhibit the activities of JAK2 and p-AKT, and suppress breast cancer proliferation (9).

Over the past few decades, gene regulatory network studies based on disease contexts and their applications have made significant progress (10,11). Recent studies are using complex machine learning models to unravel the intricate biological relationships that govern gene regulation, with promising applications in therapeutic areas such as drug target identification and personalized medicine (12–14). As the analysis of ceRNAs becomes increasingly sophisticated, the investigation of the functions and mechanisms associated with ceRNA regulatory networks is becoming more rigorous. It is therefore essential to collate and synthesize this information. Recently, several databases have been developed to house ceRNA regulatory networks and their disease associations. For instance, starBase v2.0 offers miRNA–mRNA and miRNA–lncRNA interaction networks and predicts the functions of miRNAs and other non-coding RNAs in ceRNA regulatory networks (15). LncACTdb 3.0 is designed to facilitate the individualized analysis of ceRNA networks (16). It provides ceRNA interactions and lncRNA biomarkers relevant to tumour diagnosis and therapy across various species and diseases. LnCeVar offers a detailed overview of genomic variations that disrupt the ceRNA regulatory network (17). scGRN and KnockTF 2.0 provide comprehensive single-cell gene regulatory networks between transcription factors (TFs) and their downstream target genes, and explore the knockdown/knockout effect in complex diseases (18,19). eRNAbase and SEanalysis 2.0 explore the essential regulatory role of enhancer RNAs and super-enhancers in recruiting large numbers of TFs that control the gene regulatory network and cell identity in various biological processes and diseases (20,21). Nevertheless, only a limited number of the studies that have been conducted to date provide the necessary resources and tools for the analysis of ceRNA regulation at the single-cell level. Analyses conducted at the single-cell level have the potential to facilitate the exploration of intratumoral heterogeneity and the cross-linking between cells and the tumour microenvironment (TME) (22). In comparison to traditional tissue-based analyses, single-cell level analyses offer the advantage of identifying new rare cell types and subtypes, as well as elucidating the developmental trajectory and differentiation of individual cells (23–25). Therefore, we developed LnCeCell, a comprehensive database of lncRNA-associated ceRNA networks at single-cell resolution (26). Since its initial release in 2021, there has been a marked increase in the number of functional ceRNAs and a rapid expansion of high-throughput single-cell RNA sequencing (scRNA-seq) and spatial transcriptomics sequencing (stRNA-seq) datasets. These datasets require further analysis and interpretation. Therefore, there is a great need to update LnCeCell with additional resources and enhanced features.

To meet these needs, we have updated LnCeCell to version 2.0 (LnCeCell 2.0) with significantly expanded data and improved features (Table 1). The latest update to LnCeCell 2.0 incorporates a new dataset comprising hundreds of scRNA-seq, stRNA-seq and bulk-seq datasets across 86 diseases and disease-related phenotypes, with varying clinical follow-up and treatments (such as chemotherapy, immunotherapy and targeted therapy). In addition, datasets of 80 human normal tissues/organs have been integrated. Based on these data, 836 581 cell-specific and spatial spot-specific lncRNA-associated ceRNA interactions and functional networks for 1 002 988 cells and 367 971 spatial spots have been newly identified. Following manual curation, over 15 000 experimentally supported lncRNA biomarkers (associated with cancer cell metastasis, recurrence, prognosis, circulation, drug resistance, immune response, etc.) have been collated. Furthermore, LnCeCell 2.0 offers enhanced detail regarding cell type and cell state annotation, as well as subcellular and extracellular locations of ceRNAs, through manual curation from literature and related data sources. In order to facilitate data retrieval and analysis, a panel of flexible tools (comprising 8 comprehensive analysis tools and 16 mini-analysis tools) has been developed by LnCeCell 2.0. These tools facilitate the comprehensive analysis of ceRNA distribution across diverse cellular and spatial domains, enabling the investigation of ceRNA network dynamics across cell lineages and within the TME. Collectively, the updated database is expected to facilitate the investigation of fine-tuned lncRNA-ceRNA networks with single-cell and spatial spot resolution, thereby providing insights into the regulatory mechanisms that underpin complex microbial ecosystems.

**Table 1.** The expansion of data and improvement of functions in LnCeCell 2.0

Datasets and features		LnCeCell 1.0	LnCeCell 2.0	Fold increase↑
High-throughput datasets	scRNA-seq data	40	204	5.1↑
	stRNA-seq data		53	New
	Bulk RNA data	33	110	3.33↑
	Cells	94 455	1002 988	10.62↑
	Cell types		117	New
	Spatial cells/spots		367 971	New
	Samples	10 269	20 326	1.98↑
	Diseases/phenotypes	25	86	3.44↑
	Normal tissues/organs		80	New
Gene annotation and interactions	ceRNA interactions	93 307	836 581	8.97↑
	lncRNA–miRNA interactions		41 306	New
	mRNA–miRNA interactions		45 855	New
	lncRNAs	2648	5699	2.15↑
	mRNAs	9095	19 643	2.16↑
	miRNAs		2588	New
	Diagnostics & therapy biomarkers	9306	15 489	1.66↑
Function annotation and analysis tools	Subcellular and extracellular locations	111 676	312 137	2.80↑
	Clinical samples	10 141	19 491	1.92↑
	Mini-analysis tools	7	16	2.29↑
	Comprehensive analysis tools		8	New
	Cell type annotation		√	New
	Cell trajectory analysis		√	New
	Multi-level correlation analysis		√	New
	Spatial data analysis		√	New
	Gene expression visualization		√	New
	3D spatial visualization		√	New
	Pan-cancer visualization		√	New

## Improved data expansion and new features

### Expansion of high-throughput scRNA-seq data

**Figure 1.**
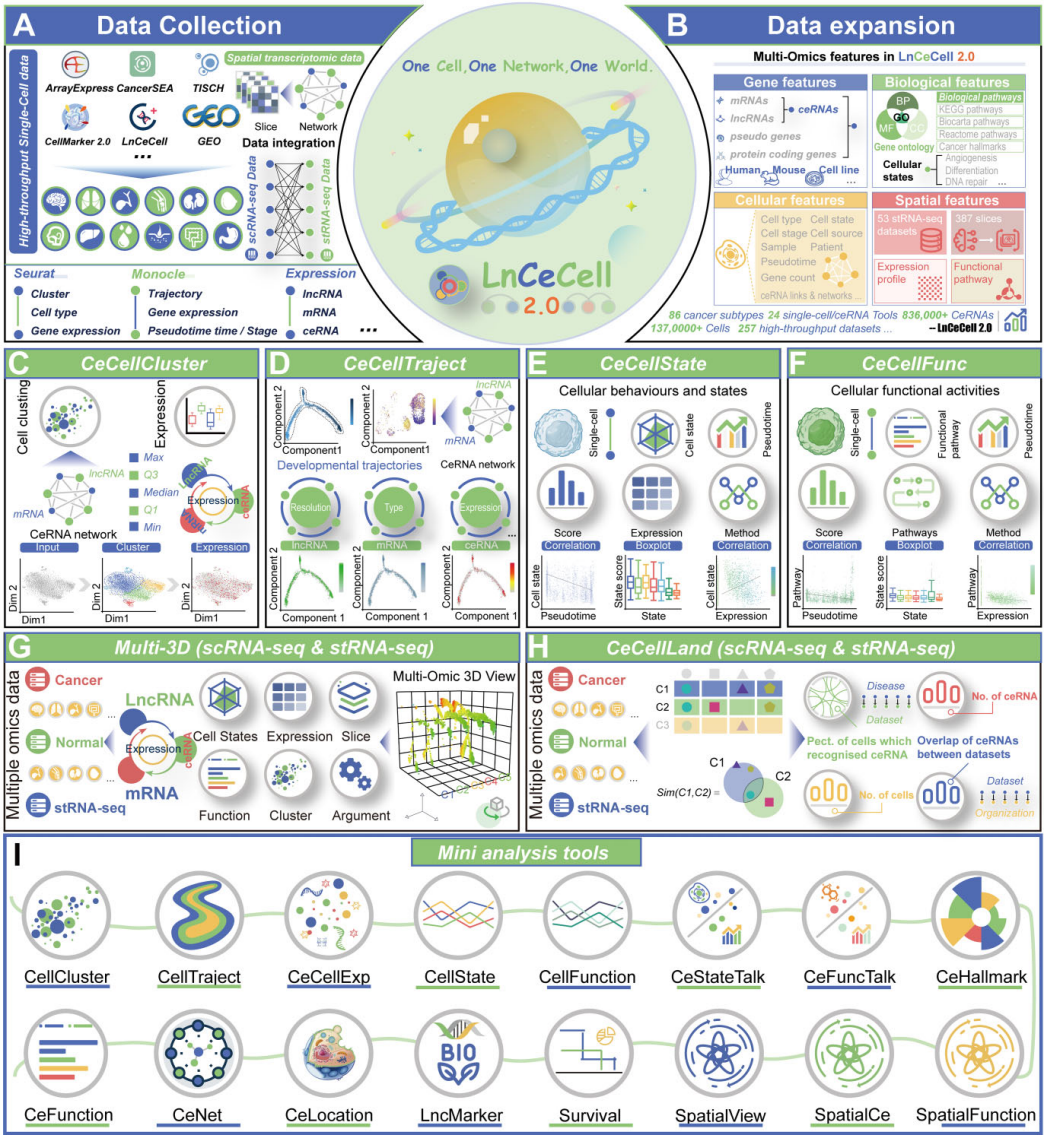
Data expansion and new features of LnCeCell 2.0. (**A**,**B**) The collection and expansion of high-throughput sequencing and manual curation datasets into the LnCeCell 2.0 database. (**C**–**I**) LnCeCell 2.0 offers a range of flexible, comprehensive and mini-analysis tools for the investigation of ceRNA-regulated mechanisms at single-cell/spot resolution.

LnCeCell 2.0 has been updated with an increased number of scRNA-seq datasets (Figure 1A and B). After data collection and pre-processing (Supplementary Methods), the current version of LnCeCell contains 204 scRNA-seq expression datasets, covering a total of 1 002 988 cells and 117 cell types. To expand the disease coverage of LnCeCell 2.0, 86 diseases and disease-related phenotypes with different clinical outcomes and treatment options (such as chemotherapy, immunotherapy and targeted therapy) were included (Supplementary Figure S1). Moreover, datasets comprising normal human organs and tissues have been collated from a previous study with the objective of determining the cell type composition of all major human organs and constructing a scheme for the human cell landscape (27). We have identified 97 gene expression profiles covering all major human organs, including both adult and fetal tissues (Supplementary Figure S2). The mean number of cells per dataset was 4916, with atherosclerosis (GSE131778) having the largest number of cells (*n* = 11 756) and melanoma (GSE157743) having the smallest number of cells (*n* = 102). For each dataset, LnCeCell 2.0 identified human and mouse gene expression profiles for different gene types, including coding genes, lncRNAs, pseudogenes and other gene types according to GENCODE gene annotations (GRCh38) (28). Finally, a total of 19 643 coding genes and 5699 non-coding genes were identified from scRNA-seq data. Compared to LnCeCell 1.0, the updated database has been significantly expanded in terms of the number of data, cells, cell types, genes and disease types (Table 1).

### New collection of high-throughput stRNA-seq data

Recent technological advances in spatial transcriptomics have made it possible to systematically measure gene expression in relation to the spatial positions of tissue, providing spatially resolved information to gain biological insight into a range of complex diseases (29). To provide initial observations of ceRNA regulation at the spatial level, LnCeCell 2.0 collected human and mouse stRNA-seq datasets by manually searching publications on PubMed and obtaining publicly available and integrated datasets from NCBI-GEO (30), EMBL-EBI (31), SpatialDB (32), SPASCER (33), Aqulia (34) and 10x Genomics (https://www.10xgenomics.com/). The current version of the LnCeCell database contains 53 spatial transcriptomics datasets covering 22 tissues and 41 diseases/phenotypes (Supplementary Figure S3). There were three main parts of the spatial omics data, including (i) the gene expression profiles across different spatial cells/spots, (ii) the spatial locations with 3D coordinates for each cell/spot and (iii) the histological images of tissue sections shown as background for gene expression. LnCeCell 2.0 measured the expression of 36 117 genes in 367 971 spatial cells/spots across 387 tissue sections and identified candidate ceRNAs for re-stratification of the TME (Supplementary Methods). The collection of the above spatial transcriptomics gene expression data by fusing with high-resolution histological images may improve the interpretability of histopathology and further help to investigate the regulatory mechanisms of ceRNA networks within the spatial organization of cells in tissues.

### Newly identified cell-specific and spatially cell/spot-specific ceRNA interactions

Based on high-throughput scRNA-seq and stRNA-seq data, LnCeCell 2.0 identified lncRNA-associated ceRNA interactions and provided a cell-specific and spatially cell/spot-specific network. The candidate ceRNAs were collected from previous databases providing potential ceRNA interactions in various diseases (15,35), and the common ceRNAs were then identified as potential candidates for regulation. We used a published method for cell-specific network construction based on probability theory to identify ceRNAs in single-cell, which assumes that ceRNA pairs may be associated in some cells but not in others (36). For each ceRNA interaction, a *P*-value and false discovery rate (FDR) were calculated to determine whether a candidate was related in a cell/spot by testing for statistical independence (Supplementary Methods, Supplementary Figure S4), and a ceRNA activity score was calculated as -log_10_(*P*-value). A total of 836 581 unique ceRNAs (FDR < 0.05) were included in the database. In order to provide more detailed and reliable information, LnCeCell 2.0 has been updated to include a greater number of experimentally verified ceRNA interactions, which have been collated from our previous studies (16,26,35). A total of 5669 ceRNA interactions were validated through the implementation of high-confidence experimentation techniques, including the utilization of luciferase reporter assays, polymerase chain reactions, western blots and other methodologies. Based on scRNA-seq data, LnCeCell 2.0 enables the investigation of ceRNA distribution across distinct cell clusters and provides a cell-specific ceRNA network, which can be employed to examine the relationships between molecular networks and cellular functions/states. Based on stRNA-seq data, LnCeCell 2.0 provides a spatial distribution of ceRNA regulations with tissue-specific positional information, which may enhance the interpretability of histopathology and its clinical utility.

### Manual curation of biomarkers, functional annotations and clinical information

In order to gain a comprehensive understanding of the roles of lncRNA-associated ceRNAs, we undertook a manual curation of their annotations in relation to cancer biomarkers, biological functions and clinical applications. As previously demonstrated (26,37), the lncRNA biomarkers related to the pathology, diagnosis and treatment were classified as autophagy, apoptosis, cell growth, circulation, drug resistance, epithelial–mesenchymal transition (EMT), immunity, metastasis, recurrence and prognosis (Supplementary Figure S5). Following manual curation (Supplementary Methods), a total of 15 489 experimentally supported lncRNA biomarkers were collated into LnCeCell 2.0. Moreover, LnCeCell 2.0 includes a list of 16 604 gene sets, encompassing a vast array of functional annotations, such as Gene Ontology (GO) terms (38), biological pathways (39), cancer cell states (40), classical cancer hallmarks (41), and subcellular and extracellular locations (42), thereby facilitating a comprehensive functional analysis. To infer the regulatory effect of ceRNA on clinical characteristics, LnCeCell 2.0 derived gene expression profiles of 19 491 cancer patients from TCGA (43) and NCBI-GEO (30) with varying clinical treatment and follow-up information. The integration of cancer biomarkers, functional annotations and clinical features into LnCeCell 2.0 will provide an enhanced functional background to study ceRNA regulatory mechanisms through pathology analysis.

### New features and enhanced web tools

Based on the theory of ‘one cell, one network, one world’, LnCeCell 2.0 has been updated with several new features to explore gene expression and ceRNA regulation at the single-cell and spatial level (Supplementary Figure S6). These features will allow us to study the distribution of ceRNAs in different cell clusters, cell types and specific histological sites, and to further explore the dynamic changes in ceRNA regulation and their correlations with cellular functions/states. A panel of 24 flexible tools (including 8 comprehensive and 16 mini-analysis tools) has been developed to investigate ceRNA-regulated mechanisms at single-cell/spot resolution (Figure 1C–I). The comprehensive analysis tools facilitate the implementation of a series of consecutive steps for integrated analysis. For example, the *CeCellCluster* and *CeCellTraject* tools perform cell cluster analysis to identify diverse cell populations with different states, and construct a cell developmental trajectory to illustrate cell-specific ceRNA network dynamics across cell lineages (Figure 1C and D). The *CeCellState* and *CeCellFunc* tools facilitate the investigation of correlation analysis among genes, ceRNAs, functions, pathways and cellular states across diverse cell populations (Figure 1E and F). The *Multi-3D* (*scRNA-seq&stRNA-seq*) tool provides web-based interfaces for the combination and visualization of multilevel features in single-cell and spatially resolved transcriptomics data (Figure 1G). The *CeCellLand* (*scRNA-seq & stRNA-seq*) tool offers a comprehensive overview of cell-specific ceRNA relationship distribution across pan-cancers and normal tissues/organs at single-cell and spatial resolution (Figure 1H). Moreover, the mini-analysis tools offer a range of rapid and user-friendly functions, including hallmark and biomarker annotation, network construction, subcellular and extracellular location mapping, survival analysis and others (Figure 1I).

## Database construction and improved user interface

LnCeCell 2.0 was employed for the management of data utilizing the MySQL software (version 5.5). The web pages were developed using Java Server Pages and deployed on the Tomcat web server (v6). A number of JavaScript plugins were employed for the creation and visualization of data tables, including jQuery.js (v1.11.3), Datatable.js (1.10.10) and ECharts.js (V4.0). All statistical analyses were conducted using the R framework (v4.2.1). The LnCeCell 2.0 database is accessible via the following link: http://bio-bigdata.hrbmu.edu.cn/LnCeCell. The version 1.0 of LnCeCell remains accessible for users who require it. To access LnCeCell 1.0, users can visit links from the LnCeCell 2.0, homepage or directly at http://bio-bigdata.hrbmu.edu.cn/LnCeCell1.0.

**Figure 2.**
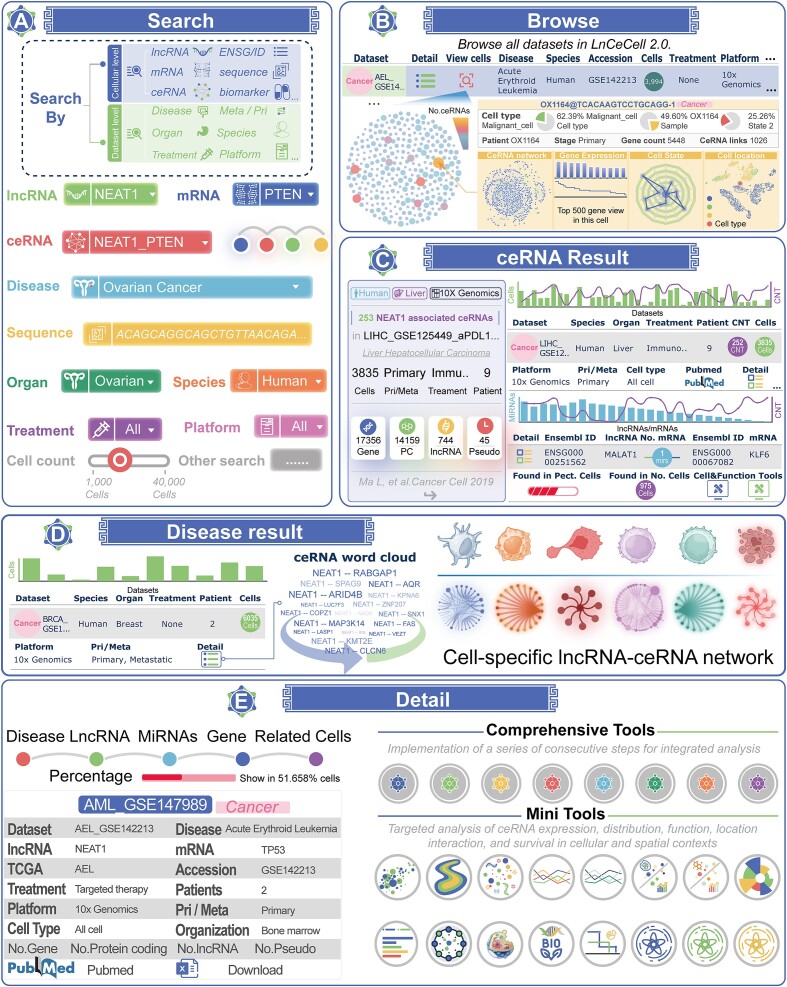
The data query workflow of LnCeCell 2.0 database. (**A**) The searching interface and options of LnCeCell 2.0 database. (**B**) The browsing interface of LnCeCell 2.0 database. (**C**) The detailed result information of ceRNAs. (**D**) The detailed result information of ceRNA-related disease. (**E**) The detailed result information of cell-specific and spatial spot-specific ceRNA interactions.

LnCeCell 2.0 offers users a convenient, user-friendly web interface that allows for searching, browsing, analysis and downloading of data (Figure 2A–E). The LnCeCell 2.0 ‘HOME’ page offers a search engine, enabling users to conduct efficient investigations of data or analyses (Supplementary Figure S7). A variety of keywords, including but not limited to lncRNAs, mRNAs, ceRNAs, diseases, organs and sequences, can be employed to search and browse the LnCeCell 2.0 database (Figure 2A and B). In order to illustrate the functionality of LnCeCell 2.0 database, we used the well-studied lncRNA NEAT1 as a case study. All data records relevant to the NEAT1 are presented as data panels and tables on the search results page (Figure 2C and D and Supplementary Figures S8 and S9). To obtain interesting records, users are able to reorder the results table in a flexible manner by clicking on the table headers. The ‘detail’ column will direct users to a page containing comprehensive information on the disease-ceRNA associations, diagnostic and treatment procedures for patients, experimental-supported annotations, the number and percentage of cells in which ceRNA can be detected and so on (Figure 2E and Supplementary Figure S10).

**Figure 3.**
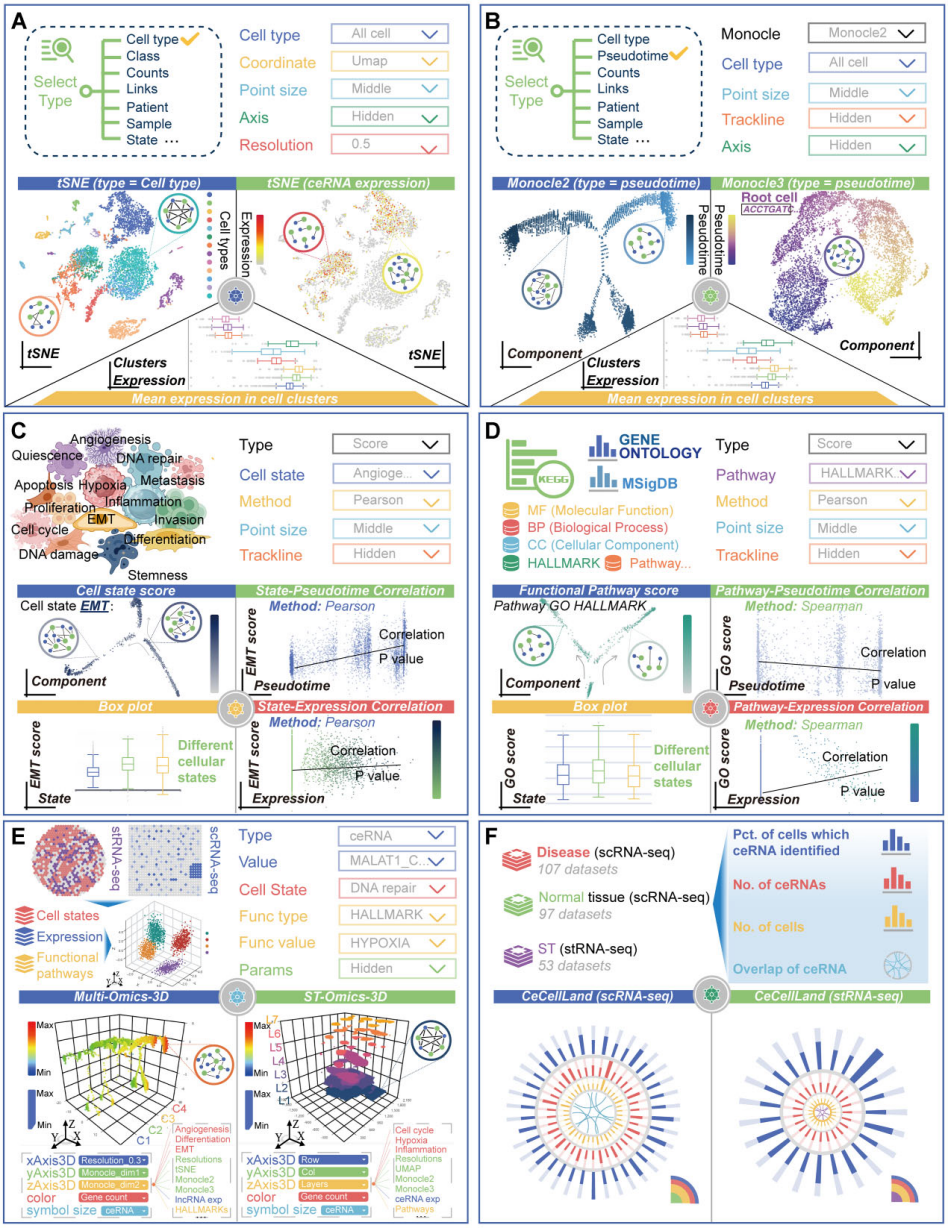
A case study for utilization of data analysis tools in LnCeCell 2.0. (**A**) The distribution of NEAT1-related ceRNA interactions across diverse cell populations. (**B**) The dynamic alteration of NEAT1-related ceRNA networks within distinct cell state lineages. (**C**, **D**) An investigation of the correlation between NEAT1-related ceRNA occurrence and cell state transitions. (**E**) The interactive 3D interfaces enable the investigation of the complex interplay between NEAT1-related ceRNA networks and functions based on scRNA-seq and stRNA-seq data. (**F**) A comprehensive landscape of ceRNA distribution across a range of pan-cancers and normal tissues/organs.

Moreover, a panel of online tools for the study of ceRNA-regulated mechanisms at the single-cell/spot resolution level has been developed (Figure 3A–F). In accordance with the principles of intracellular gene expression, the cells can be classified into different populations, which can then be visualized on the basis of their cell type, gene content, ceRNA profiles, patient identity, disease stage and other relevant characteristics. This is achieved by supplementing the classification with the coordinates of the cellular distribution. The comprehensive mapping of NEAT1 expression and its associated ceRNA interactions in diverse cell populations was investigated using the *CeCellCluster* tool (Figure 3A and Supplementary Figure S11). In order to gain insight into the dynamics of NEAT1-related ceRNA networks and their associations within different cell state lineages, a cell developmental trajectory was constructed and illustrated using the *CeCellTraject* tool (Figure 3B and Supplementary Figure S12). To elucidate the phenotypic and functional heterogeneity of the TME, we employed the *CeCellState* and *CeCellFunc* tools of LnCeCell 2.0 to evaluate the cell state transitions based on different functional contexts (Figure 3C and D). For instance, our findings revealed the existence of diverse cellular states with elevated EMT activity scores in acute erythroid leukaemia (GSE142213). Additionally, a positive correlation was identified between NEAT1 expression and EMT score (Figure 3C and Supplementary Figure S13), suggesting that NEAT1 plays a potential role in regulating EMT activity (44). By utilizing the distribution map of distinct cell clusters or developmental trajectories, users are able to construct a cell-specific network by clicking on any cell, thereby enabling a side-by-side comparison of ceRNA regulations within diverse cellular contexts (Figure 3A–D). The *Multi-3D* (*scRNA-seq & stRNA-seq*) tool enables the examination of the complex interplay between NEAT1-associated ceRNA networks and functions (such as GO terms, biological pathways, cancer hallmarks, etc.) that influence individual disease pathology and cell fate (Figure 3E and Supplementary Figure S14). This analysis is presented in an interactive 3D format, offering a comprehensive visual representation. In order to provide a more global perspective of the ceRNA regulatory relationships associated with NEAT1, the *CeCellLand* (*scRNA-seq & stRNA-seq*) tool offers a comprehensive landscape of ceRNA distribution across a range of pan-cancers and normal tissues/organs (Figure 3F and Supplementary Figure S15). In summary, the web-based tools of LnCeCell 2.0 provide an efficient means of analysing NEAT1-regulating mechanisms, offering detailed insights for integration, comparison and visualization based on single-cell and spatially resolved transcriptomics data.

## Conclusions and future development

Since the development of LnCeCell (version 1.0) in 2021, there has been a notable increase in the number of functional ceRNAs and a rapid expansion of high-throughput single-cell and spatial transcriptomics datasets. The advancement of single-cell and spatially resolved transcriptomics techniques has resulted in the accumulation of complex datasets that encompass both cellular-specific and spatial histological position information. Further analysis and interpretation of these datasets is required. Consequently, there is a pressing need to update LnCeCell with additional resources and enhanced analysis tools. To meet these needs, we have updated LnCeCell to version 2.0 (LnCeCell 2.0) with significantly expanded data and improved features. The latest update to LnCeCell 2.0 incorporates a new dataset comprising hundreds of scRNA-seq, stRNA-seq and bulk-seq datasets across 86 disease-related phenotypes and 80 human normal organs/tissues, with varying clinical follow-up and treatment options. The collection of the above spatial transcriptomics gene expression data by fusing with high-resolution histological images may improve the interpretability of histopathology and further help to investigate the regulatory mechanisms of ceRNA networks within the spatial organization of cells in tissues. A total of 836 581 cell-specific and spatial spot-specific ceRNA interactions and functional networks for 1 002 988 cells and 367 971 spatial spots have been newly identified. More than 15 000 experimentally supported lncRNA biomarkers have been collated through manual curation. Moreover, LnCeCell 2.0 provides a more comprehensive account of cell type and cell state annotation, in addition to the subcellular localizations of ceRNAs. This is achieved through the manual curation of information from a range of published sources and other datasets. In order to facilitate the retrieval and analysis of data, a panel of flexible tools has been developed by LnCeCell 2.0. This comprises 8 comprehensive analysis tools and 16 mini-analysis tools. The comprehensive analysis tools facilitate the comprehensive analysis of ceRNA distribution across diverse cellular and spatial domains, thereby enabling the investigation of ceRNA network dynamics across cell lineages and within the TME. In addition, the mini-analysis tools offer a range of user-friendly functions, including hallmark and biomarker annotation, network construction, subcellular and extracellular location mapping, survival analysis and others, which can be employed as a fast and easy-to-use approach. As technology advances, an increasing volume of biomedical big data will be generated, thereby enhancing the comprehensiveness of our research and improving the accuracy of our results. In the future, the LnCeCell database will be maintained and updated on a continual basis with the addition of further datasets and the implementation of enhanced services. Furthermore, the integration of single-cell and spatial transcriptomics with more reliable approaches will be facilitated, thereby improving the interpretability of histopathology and its utilization in clinical decision-making processes to guide treatment and inform prognosis.

## Supplementary Material

gkae947_Supplemental_File

## Data Availability

All the data could be downloaded from http://bio-bigdata.hrbmu.edu.cn/LnCeCell.
