# Supplementary material for: LnCeCell 2.0: an updated resource for lncRNA-associated ceRNA networks and web tools based on single-cell and spatial transcriptomics sequencing data
Source: Nucleic Acids Res. 2024 Oct 29;53(D1):D107–15. doi: 10.1093/nar/gkae947 (PMC11701739; doi:10.1093/nar/gkae947)
Supplement: gkae947_Supplemental_File [file gkae947_supplemental_file.pdf]

Supplementary Information for

LnCeCell 2.0: an updated resource for lncRNA-associated ceRNA networks and web tools based on single-cell and spatial transcriptomics sequencing data

This file includes:

Supplementary Methods

Supplementary Figures (Figure S1-S15)

Supplementary Methods

High-throughput sequencing data collection and processing

LnCeCell 2.0 collected high-throughput single-cell RNA sequencing (scRNA-seq) and spatial transcriptomics sequencing (stRNA-seq) datasets by manually searching publications on PubMed and retrieving data from NCBI-GEO (https://www.ncbi.nlm.nih.gov/geo/) (1), EMBL-EBI (https://www.ebi.ac.uk/) (2), ArrayExpress (https://www.ebi.ac.uk/arrayexpress/) (3), TISCH (http://tisch.comp-genomics.org/home) (4), SpatialDB (http://spatialomics.org/SpatialDB/) (5), SPASCER (https://ccsm.uth.edu/SPASCER/index.html) (6), Aquila (https://aquila.cheunglab.org/) (7), and 10xGenomics (https://www.10xgenomics.com/). Moreover, datasets comprising normal human organs and tissues have been collated from NCBI-GEO with accession of GSE134355. Only datasets containing more than 100 single cells or spatial spots/cells were included. For each dataset, corresponding metadata, including sample ID, organ/tissue origin, clinical treatment, biosample groups, primary/metastatic sites, and cell types, were integrated into LnCeCell 2.0. Gene annotation files were obtained from the GENCODE database (https://www.gencodegenes.org/, release 41, GRCh38) (8) to identify various gene types such as protein-coding genes, long non-coding RNAs (lncRNAs), pseudogenes, etc. Cells/spots expressing fewer than 1,000 genes were excluded. Genes with detectable expression in at least 1% of cells/spots were retained. For scRNA-seq data, quality control (QC) and data normalization were performed using the R package Seurat (v4.3.0, https://satijalab.org/seurat/) (9). For stRNA-seq data, the Python package Scanpy (v1.9.3) (10) and the R package Seurat (v4.3.0) were used for QC and normalization analysis. Specifically, Scanpy (v1.9.3) processed spatial transcriptomic datasets in the h5ad format. The 'SCTransform' function in

Seurat (v4.3.0) normalized the gene expression matrix, converted count values to TPM values, and performed logarithmic conversion on the final results. After raw data QC and normalization, 257 scRNA-seq and stRNA-seq datasets encompassing 86 diseases/phenotypes and 80 human normal tissues were collected.

Cell clustering analysis

We performed unsupervised clustering analysis of gene expression profiles using Seurat (v4.3.0). Principal component analysis (PCA) was conducted, and a significant subset of principal components (PCs) was chosen for further classification using Seurat's JackStraw and ElbowPlot functions. The FindClusters, RunTSNE, and RunUMAP functions were used for cell clustering and visualization, respectively. The resolution parameter of the FindClusters function was adjusted from 0.1 to 0.9 (with an interval of 0.1) to produce clustering results at various resolutions, with higher resolution values corresponding to more cell clusters.

Cell type annotation

LnCeCell 2.0 performed cell type annotation using two strategies: *(i)* utilizing the original cell-type annotation provided by the data source, and *(ii)* applying the CELLiD method described by DISCO to annotate different cell types (11). In this step, cell types for each cluster were determined using reference cell type marker genes and the CELLiD R code (https://github.com/JinmiaoChenLab/DISCO_manuscript/blob/master/CELLiD.R). Cell marker annotations were collected and combined from DISCO and CellMarker 2.0 (12). DISCO is a database of deeply integrated scRNA-seq data covering 107 tissues/cell lines/organoids and

158 diseases, while CellMarker 2.0 provides manually curated markers of diverse cell types. We integrated markers from both databases for comprehensive cell type annotations. The comprehensive cell type annotation reference (including normal cells, malignant cells, and disease cells) was used as input for CELLiD for each cell cluster.

Cell development trajectories construction

The R packages Monocle 2 (v2.18.0) (13) and Monocle 3 (v1.2.9) (14) were used to calculate pseudotime, states and further construct cell development trajectories. Monocle 2, which works well with both relative expression data and count-based measures, was used with gene counts as the input matrix. The expressionFamily parameter was set as negbinomial.size() to specify the negative binomial distribution. The processed data from Seurat, containing quality filtered cells and metadata, were used in Monocle 2. Genes with an average expression greater than 0.1 were used for PCA, and the top 20 principal components were used for cell clustering. Differentially expressed genes (q-value < 0.01) were screened for cell sorting. The DDRTree algorithm was used to reduce dimensionality for trajectory visualization in 2D space. Monocle 3 (v1.2.9) introduced UMAP for dimensional reduction, which better reflects high-dimensional data. Unlike Monocle 2's DDRTree-based method that assumes a single tree-like trajectory structure, Monocle 3 can learn multiple, disjoint graphs. The top 20 principal components from PCA were used for cell clustering, and featured genes (q-value < 0.01) were selected for cell ordering. Pseudotime trajectories were visualized in the 2D space of UMAP. LnCeCell 2.0 provided trajectory analyses using both Monocle 2 and Monocle 3 for various cell types, such as malignant cells, immune cells, and stromal cells.

Functional annotation data collection

LnCeCell 2.0 includes 16,604 gene sets encompassing various functional annotations to dissect the functional activation status and state transitions of individual cellular populations. These annotations include Gene Ontology (GO) terms (15), biological pathways (16), cancer cell states (17), classical cancer hallmarks (18), and subcellular and extracellular locations (19). The gene set variation analysis (GSVA) method (20) was used to evaluate cellular functional activation status and states in each dataset. For GO annotation, LnCeCell 2.0 collected 7,658 biological process (BP) gene sets, 1,738 molecular function (MF) gene sets, and 1,006 cellular component (CC) gene sets. For pathway annotation, a total of 2,982 biological pathway gene sets from Kyoto Encyclopedia of Genes and Genomes (KEGG), BioCarta, Reactome, and other databases were collected. Additionally, LnCeCell 2.0 includes 10 classic cancer hallmark gene sets representing well-defined biological processes, such as self-sufficiency in growth signals, insensitivity to antigrowth signals, evasion of apoptosis, limitless replicative potential, sustained angiogenesis, tissue invasion and metastasis, genome instability and mutation, tumor-promoting inflammation, reprogramming energy metabolism, and evasion of immune detection. Characteristic gene sets for 14 functional states of cancer cells (e.g., stemness, invasion, metastasis, proliferation, EMT, angiogenesis, apoptosis, cell cycle, differentiation, DNA damage, DNA repair, hypoxia, inflammation, and quiescence) were also included from CancerSEA (http://biocc.hrbmu.edu.cn/CancerSEA) (17).

Construction of single cell ceRNA networks

The candidate ceRNA pairs were collected from two databases: starBase v2.0 (21) and LncACTdb 2.0 (22). The common ceRNA interactions were then identified as potential candidates for regulation. A total of 108,668 candidate ceRNA regulations were collated. To ascertain whether these ceRNA pairs were associated with each other in a single cell, we employed a published method for cell-specific network construction based on probability theory to identify ceRNA networks in single cells (Figure S4) (23). It is hypothesised that a ceRNA pair may exhibit an association in some cells but not in others due to differences in cell type.

The statistical independence of the candidate ceRNA expression values in the same cell was tested to determine whether lncRNAs and mRNAs were related in a cell. For a ceRNA pair of x (mRNA) and y (lncRNA) in cell k, the following statistic was calculated:

$$\rho_{xy}{}^{k} = \frac{\sqrt{n-1} \bullet \left(n \bullet n_{xy}{}^{k} - n_{x}{}^{k} n_{y}{}^{k}\right)}{\sqrt{n_{x}{}^{k} n_{y}{}^{k} \left(n - n_{x}{}^{k}\right)\left(n - n_{y}{}^{k}\right)}} \qquad (1)$$

where n indicates the total number of cells. The $n_{\mathrm{x}}{}^{(k)}$ and $n_{y}{}^{(k)}$ are predetermined integers and were set as $n_{\mathrm{x}}{}^{(k)} = n_{\mathrm{y}}{}^{(k)} = 0.1n$. We draw the first two boxes near x_k and y_k, based on the predetermined $n_{\mathrm{x}}{}^{(k)}$ and $n_{y}{}^{(k)}$, and then we have the third box, which is simply the intersection of the previous two boxes (Figure S4). Thus, we can obtain the value of $n_{xy}{}^{(k)}$ by counting the plots in the third box.

If x and y are independent of each other, the statistic in question will follow a standard normal distribution. In this case, the mean value and variance for the n cells are 0 and 1, respectively. Consequently, the significance of the x, y correlation can be determined using this statistic.

$edge_{xy}{}^{(k)}$ The value is set to 1 in the network of cell k, with a false discovery rate (FDR) of

less than 0.05. CeRNA pairs meeting the FDR < 0.05 threshold were retained for network construction in a single cell. The algorithm requires both mRNA and lncRNA expression profiles for scRNA-seq and stRNA-seq datasets, with a minimum of 100 cells/spots. However, there is no strict requirement for the data type of scRNA-seq array. This method is not sensitive to the normalisation method and is suitable for various types of gene expression matrices.

In scRNA-seq and stRNA-seq data, the statistic may result in zero due to experimental errors, rendering it meaningless in the context of biological analysis and potentially leading to errors in the data interpretation. Consequently, we adopt the following approach to address the issue of zeros (23): *(i)* If we cannot distinguish whether or not the zeros result from zero expression or the experimental errors, $edge_{xy}^{(k)}$ is set to 0 when $x_k = 0$ or $y_k = 0$ without the consideration of the statistic. *(ii)* If we know that the zeros result from the zero expression, $edge_{xy}^{(k)}$ is determined by the statistic.

Functional analysis of lncRNA-associated ceRNAs

The CeCellFunc and CeCellState tools were developed as part of LnCeCell 2.0 with the objective of performing functional analyses of ceRNAs on the basis of a "guilt-by-association" strategy. In the case of lncRNAs, the corresponding downstream mRNA targets were utilised to perform a function enrichment analysis. LnCeCell 2.0 employs a hypergeometric test for the evaluation of significant enrichment in distinct functional contexts. If there are a total of *N* genes in the genome, of which *S* is involved in the gene set under investigation, and there are a total of *M* interesting target genes for analysis, of which *x* are involved with the same function,

then the P value can be calculated as:

$$P = 1 - \sum_{t=0}^{x} \frac{\binom{S}{t}\binom{N-S}{M-t}}{\binom{N}{M}} \qquad (2)$$

Significantly enriched functions were defined at a level of P < 0.05 and were further illustrated as a bar graph of the –log10(P) values.

Survival analysis of ceRNA regulations

The LnCeCell-Survival tool performs Cox regression analyses and provides Kaplan-Meier survival curves for competing lncRNAs, mRNAs, and their contribution to the ceRNA networks. LnCeCell2.0 derives clinical follow-up information of 19,491 patients from TCGA and performs a univariate Cox regression analysis to evaluate the association between survival state and the expression level of each lncRNA-mRNA member in a ceRNA interaction. A risk score model was developed to evaluate the association between survival and expression in a certain cancer. This model takes into account both the strength and the positive or negative association between each competing RNA and the probability of survival (22). A risk score was calculated for each patient by means of a linear combination of ceRNA expression values, weighted by the corresponding Cox regression coefficients:

$$Risk\ score = \sum_{i=1}^{n} \beta_i Exp(c_i) \qquad (3)$$

where β_i is the Cox regression coefficient of an lncRNA, miRNA, or mRNA in a ceRNA interaction (indicated as c_i), *n* is the number of competing RNAs (n=3 in this study), and $Exp(c_i)$ is the expression value of competing RNA c_i in the corresponding sample. The median and mean risk scores were used to divide the samples into high - and low-risk groups.

Database construction

LnCeCell 2.0 is available for free access at http://bio-bigdata.hrbmu.edu.cn/LnCeCell/. The online web server of LnCeCell 2.0 was constructed using Java Server Pages (JSP) and deployed on Tomcat software (v6, https://tomcat.apache.org/). The web pages were created using HyperText Markup Language (HTML) and controlled by Java programs (https://www.oracle.com/java/). All datasets of LnCeCell 2.0 were documented and managed using the MySQL database server (version 5.5, http://www.mysql.com). A number of JavaScript packages were implemented for the generation of result data and the visualisation of multi-level data cross-talk. JQuery (version 1.11.3, https://code.jquery.com) was employed for the control of the web page style. The data result tables were generated using DataTables.js (v1.10.10, http://www.datatables.club/). ECharts.js (v4.0, https://echarts.apache.org/) was employed for data visualisation. All data processing and statistical analyses were conducted using R software (v4.2.1, https://cloud.r-project.org/).

References of Supplementary Methods

1. Barrett, T., Wilhite, S.E., Ledoux, P., Evangelista, C., Kim, I.F., Tomashevsky, M., Marshall, K.A., Phillippy, K.H., Sherman, P.M., Holko, M. *et al.* (2013) NCBI GEO: archive for functional genomics data sets--update. *Nucleic Acids Res*, **41**, D991-995.
2. Thakur, M., Bateman, A., Brooksbank, C., Freeberg, M., Harrison, M., Hartley, M., Keane, T., Kleywegt, G., Leach, A., Levchenko, M. *et al.* (2023) EMBL's European Bioinformatics Institute (EMBL-EBI) in 2022. *Nucleic Acids Res*, **51**, D9-D17.
3. Athar, A., Fullgrabe, A., George, N., Iqbal, H., Huerta, L., Ali, A., Snow, C., Fonseca, N.A., Petryszak, R., Papatheodorou, I. *et al.* (2019) ArrayExpress update - from bulk to single-cell expression data. *Nucleic Acids Res*, **47**, D711-D715.
4. Sun, D., Wang, J., Han, Y., Dong, X., Ge, J., Zheng, R., Shi, X., Wang, B., Li, Z., Ren, P. *et al.* (2021) TISCH: a comprehensive web resource enabling interactive single-cell transcriptome visualization of tumor microenvironment. *Nucleic Acids Res*, **49**, D1420-D1430.
5. Fan, Z., Chen, R. and Chen, X. (2020) SpatialDB: a database for spatially resolved transcriptomes. *Nucleic Acids Res*, **48**, D233-D237.
6. Fan, Z., Luo, Y., Lu, H., Wang, T., Feng, Y., Zhao, W., Kim, P. and Zhou, X. (2023) SPASCER: spatial transcriptomics annotation at single-cell resolution. *Nucleic Acids Res*, **51**, D1138-D1149.
7. Zheng, Y., Chen, Y., Ding, X., Wong, K.H. and Cheung, E. (2023) Aquila: a spatial omics database and analysis platform. *Nucleic Acids Res*, **51**, D827-D834.
8. Frankish, A., Carbonell-Sala, S., Diekhans, M., Jungreis, I., Loveland, J.E., Mudge, J.M., Sisu, C., Wright, J.C., Arnan, C., Barnes, I. *et al.* (2023) GENCODE: reference annotation for the human and mouse genomes in 2023. *Nucleic Acids Res*, **51**, D942-D949.
9. Satija, R., Farrell, J.A., Gennert, D., Schier, A.F. and Regev, A. (2015) Spatial reconstruction of single-cell gene expression data. *Nat Biotechnol*, **33**, 495-502.
10. Wolf, F.A., Angerer, P. and Theis, F.J. (2018) SCANPY: large-scale single-cell gene expression data analysis. *Genome Biol*, **19**, 15.
11. Li, M., Zhang, X., Ang, K.S., Ling, J., Sethi, R., Lee, N.Y.S., Ginhoux, F. and Chen, J. (2022) DISCO: a database of Deeply Integrated human Single-Cell Omics data. *Nucleic Acids Res*, **50**, D596-D602.
12. Hu, C., Li, T., Xu, Y., Zhang, X., Li, F., Bai, J., Chen, J., Jiang, W., Yang, K., Ou, Q. *et al.* (2023) CellMarker 2.0: an updated database of manually curated cell markers in human/mouse and web tools based on scRNA-seq data. *Nucleic Acids Res*, **51**, D870-D876.
13. Qiu, X., Mao, Q., Tang, Y., Wang, L., Chawla, R., Pliner, H.A. and Trapnell, C. (2017) Reversed graph embedding resolves complex single-cell trajectories. *Nat Methods*, **14**, 979-982.
14. Cao, J., Spielmann, M., Qiu, X., Huang, X., Ibrahim, D.M., Hill, A.J., Zhang, F., Mundlos, S., Christiansen, L., Steemers, F.J. *et al.* (2019) The single-cell transcriptional landscape of mammalian organogenesis. *Nature*, **566**, 496-502.
15. Gene Ontology, C. (2021) The Gene Ontology resource: enriching a GOld mine. *Nucleic Acids Res*, **49**, D325-D334.
16. Castanza, A.S., Recla, J.M., Eby, D., Thorvaldsdottir, H., Bult, C.J. and Mesirov, J.P. (2023) Extending support for mouse data in the Molecular Signatures Database (MSigDB). *Nat Methods*, **20**, 1619-1620.
17. Yuan, H., Yan, M., Zhang, G., Liu, W., Deng, C., Liao, G., Xu, L., Luo, T., Yan, H., Long, Z. *et al.*

(2019) CancerSEA: a cancer single-cell state atlas. *Nucleic Acids Res*, **47**, D900-D908.

18. Hanahan, D. and Weinberg, R.A. (2011) Hallmarks of cancer: the next generation. *Cell*, **144**, 646-674.
19. Qi, Y., Xu, R., Song, C., Hao, M., Gao, Y., Xin, M., Liu, Q., Chen, H., Wu, X., Sun, R. *et al.* (2024) A comprehensive database of exosome molecular biomarkers and disease-gene associations. *Sci Data*, **11**, 210.
20. Hanzelmann, S., Castelo, R. and Guinney, J. (2013) GSVA: gene set variation analysis for microarray and RNA-seq data. *BMC Bioinformatics*, **14**, 7.
21. Li, J.H., Liu, S., Zhou, H., Qu, L.H. and Yang, J.H. (2014) starBase v2.0: decoding miRNA-ceRNA, miRNA-ncRNA and protein-RNA interaction networks from large-scale CLIP-Seq data. *Nucleic Acids Res*, **42**, D92-97.
22. Wang, P., Li, X., Gao, Y., Guo, Q., Wang, Y., Fang, Y., Ma, X., Zhi, H., Zhou, D., Shen, W. *et al.* (2019) LncACTdb 2.0: an updated database of experimentally supported ceRNA interactions curated from low- and high-throughput experiments. *Nucleic Acids Res*, **47**, D121-D127.
23. Dai, H., Li, L., Zeng, T. and Chen, L. (2019) Cell-specific network constructed by single-cell RNA sequencing data. *Nucleic Acids Res*, **47**, e62.

Supplementary Figures

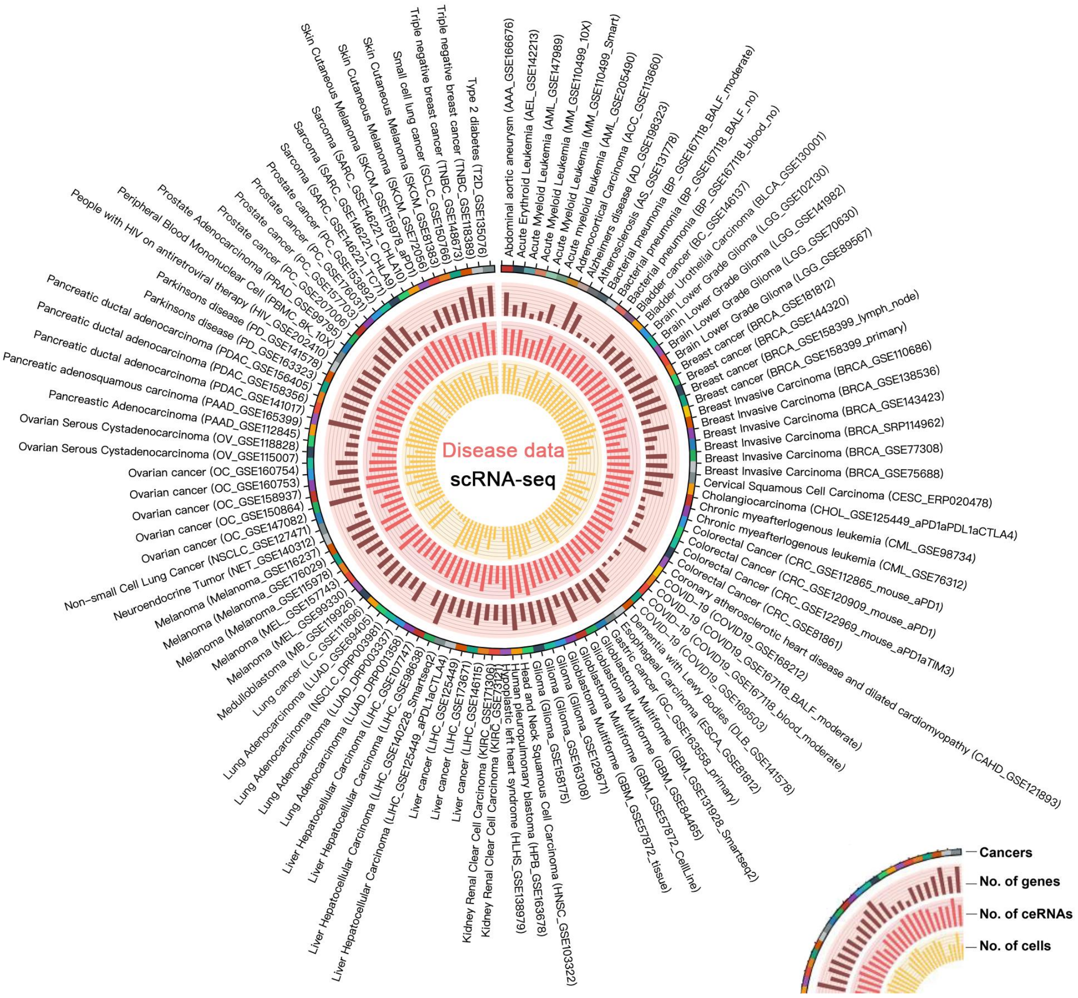

Figure S1. An overview of disease-related scRNA-seq datasets with various clinical outcomes and treatment options available in LnCeCell 2.0.

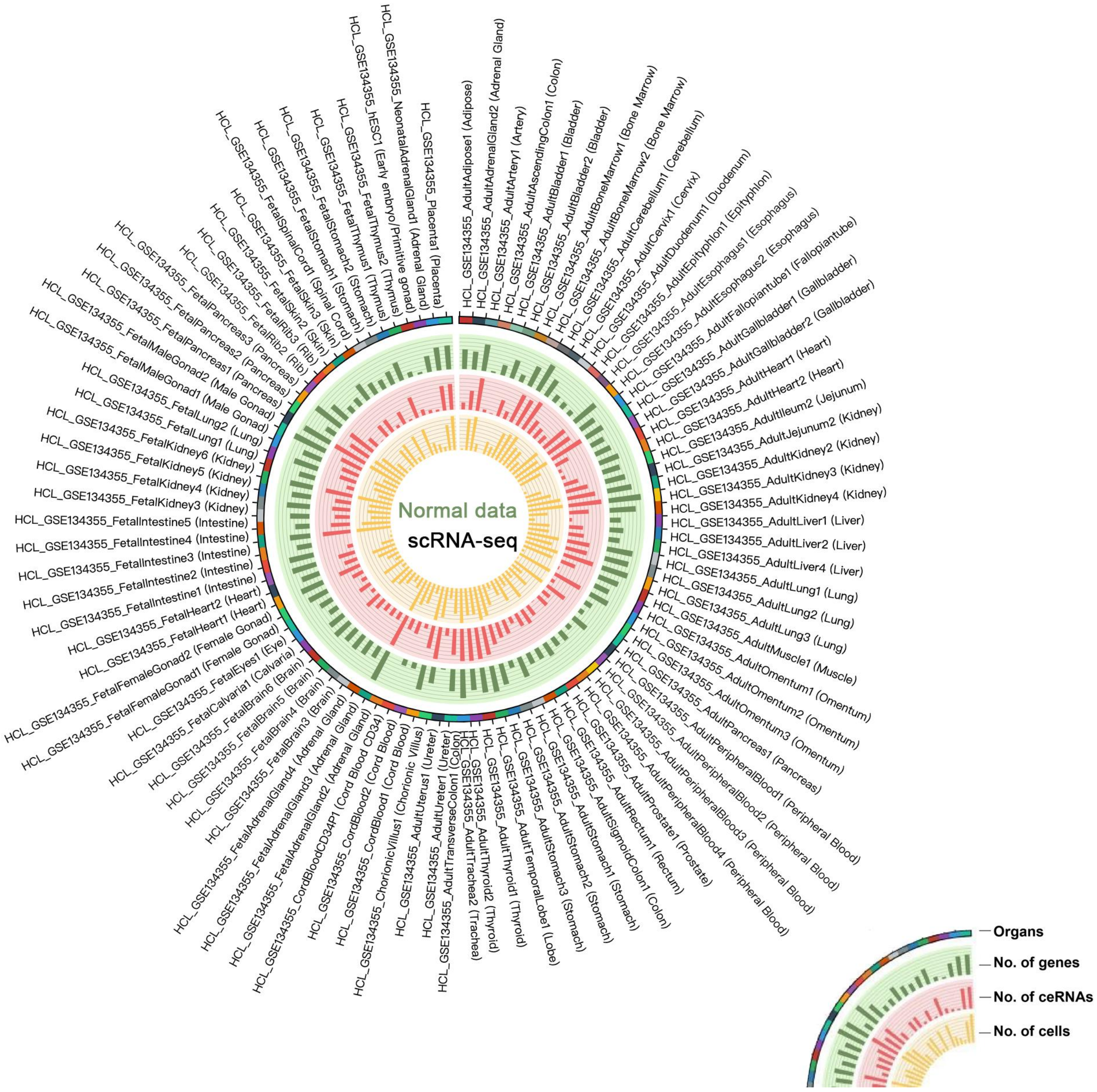

Figure S2. An overview of the datasets comprising normal human organs and tissues in LnCeCell 2.0.

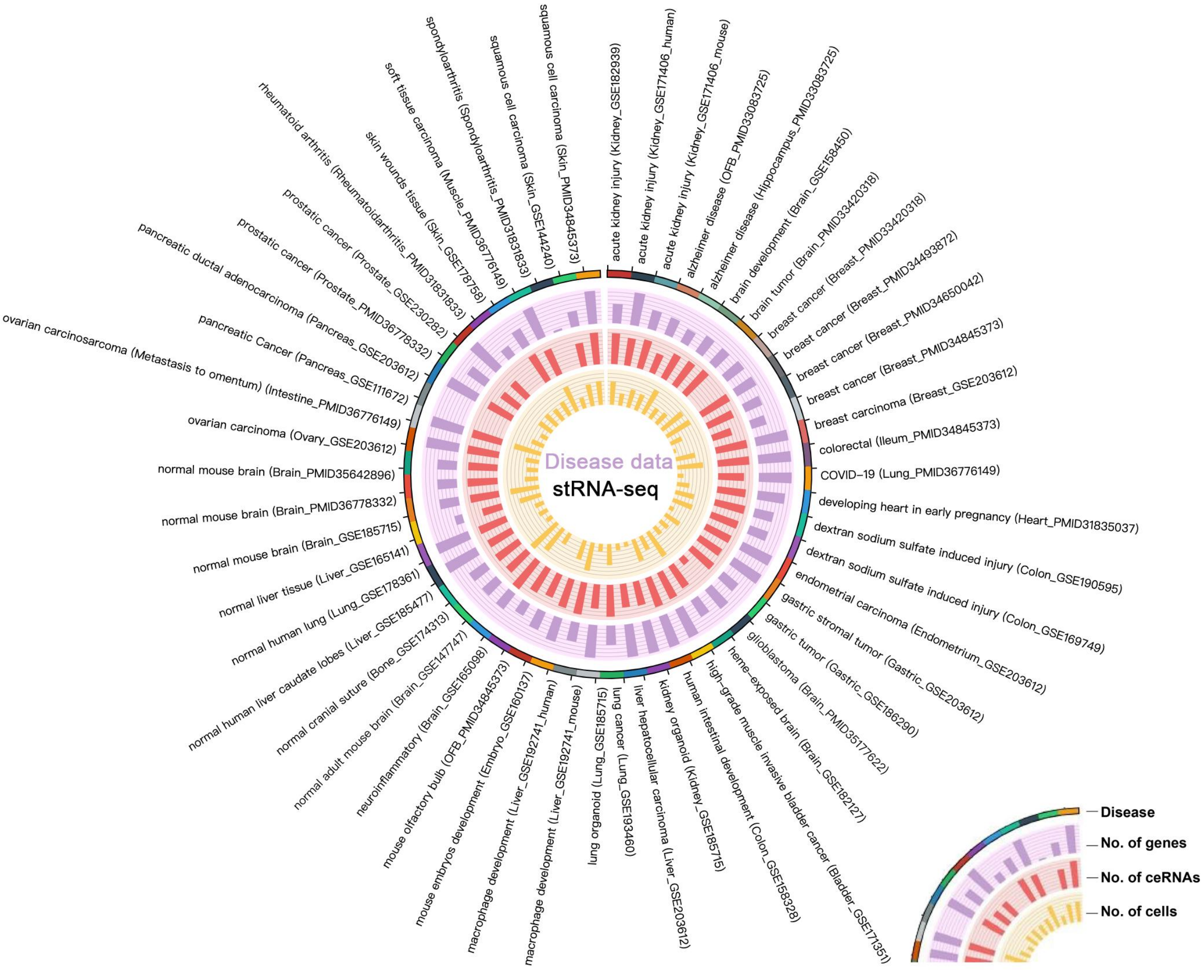

Figure S3. An overview of spatial transcriptomic datasets in LnCeCell 2.0, covering 22 different tissues and 41 distinct diseases or phenotypes.

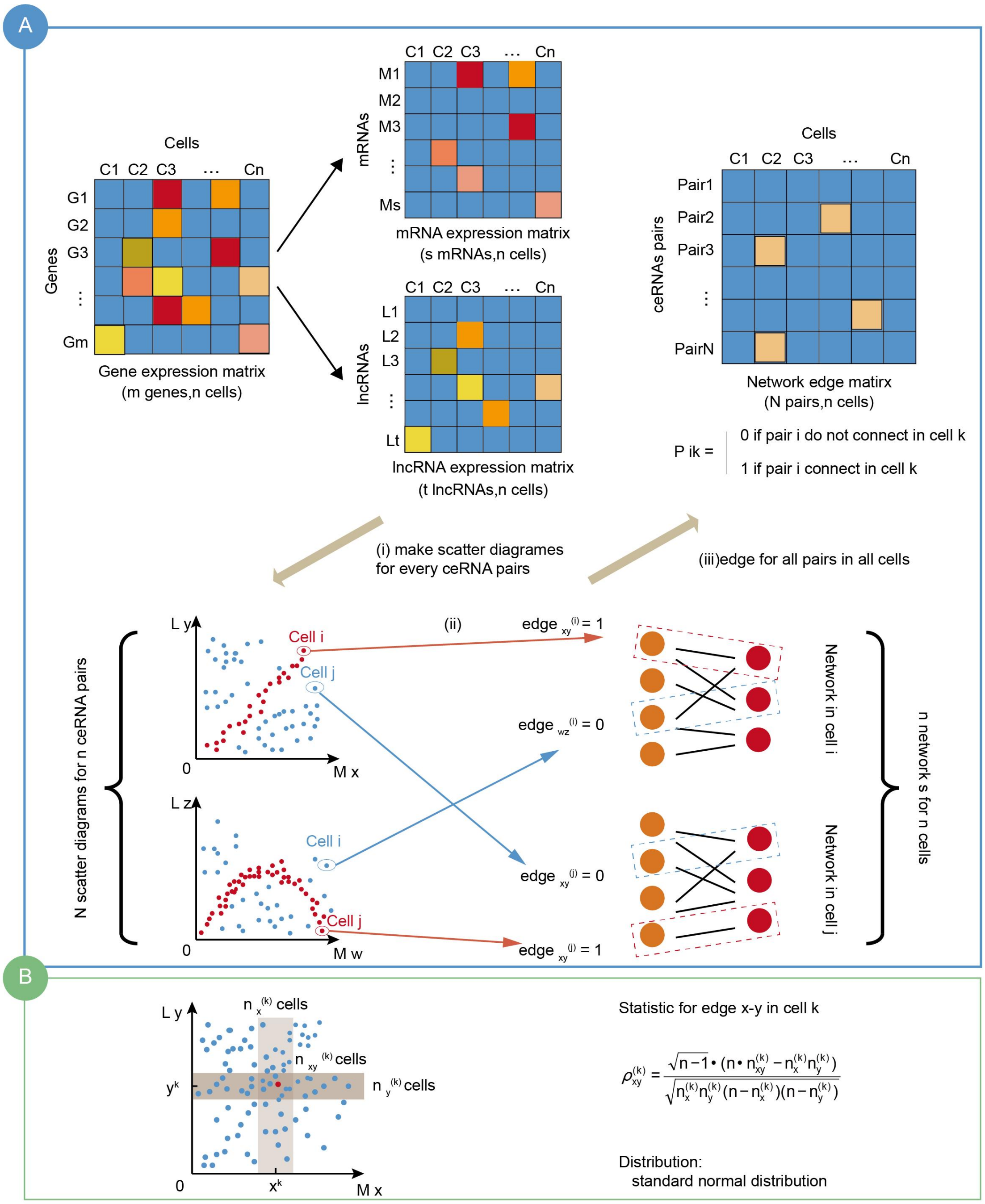

$$\rho_{xy}^{(k)} = \frac{\sqrt{n-1}\cdot(n\cdot n_{xy}^{(k)} - n_x^{(k)} n_y^{(k)})}{\sqrt{n_x^{(k)} n_y^{(k)} (n-n_x^{(k)})(n-n_y^{(k)})}}$$

Figure S4. A statistical model is employed to calculate cell-specific or spot-specific ceRNA networks. **(A)** The construction of single cell and spatial spot-specific ceRNA networks. (i) Scatter diagrams for every ceRNA pair, wherein each point represents a cell, and x- and y-values are the expression values of mRNA and lncRNA respectively in the n cells. Then N ceRNA pairs lead to N scatter diagrams. (ii) In the scatter diagram of mRNA (x) and lncRNA (y), a red plot signifies an edge between x and y in the cell-specific network, based on our statistical model, and a blue plot signifies no edge. We can then construct n cell-specific networks corresponding to n cells. (iii) We get the ceRNA occurrence profile, comprised of N

rows and n columns. If pair i is connected in cell k, $P_{ik} = 1$; or else, $P_{ik} = 0$. **(B)** The statistical model for the edge between mRNA: x and lncRNA: y. Near the plot or cell k, the light and medium grey boxes represent the neighbourhood of x_k and y_k respectively. The intersection of the two boxes is the dark grey box, which represents the neighbourhood of (x_k, y_k) . The number of plots in the light, medium and dark grey boxes is $n_{\mathrm{x}}^{(k)}$, $n_y^{(k)}$ and $n_{xy}^{(k)}$ respectively. The statistic is designated as $\rho_{xy}^{(k)}$ .If x and y are independent of each other, the statistic follows standard normal distribution. If the statistic $\rho_{xy}^{(k)}$ is significantly larger, there is an edge between x and y in cell k; otherwise there is no edge.

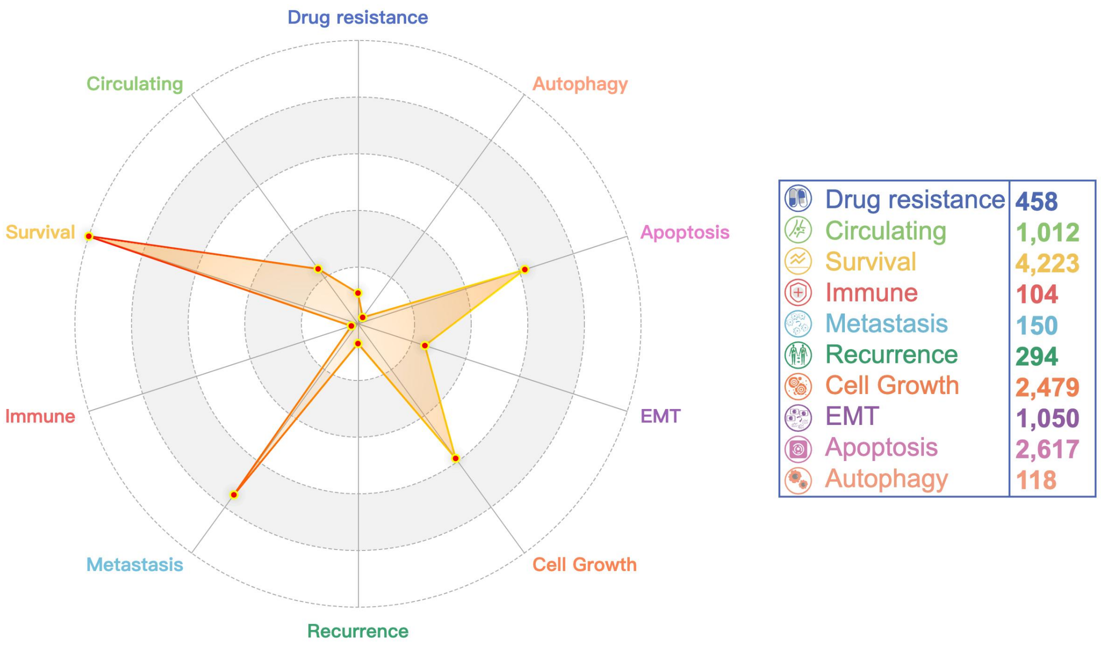

Figure S5. An overview of lncRNA biomarkers curated manually in LnCeCell 2.0.

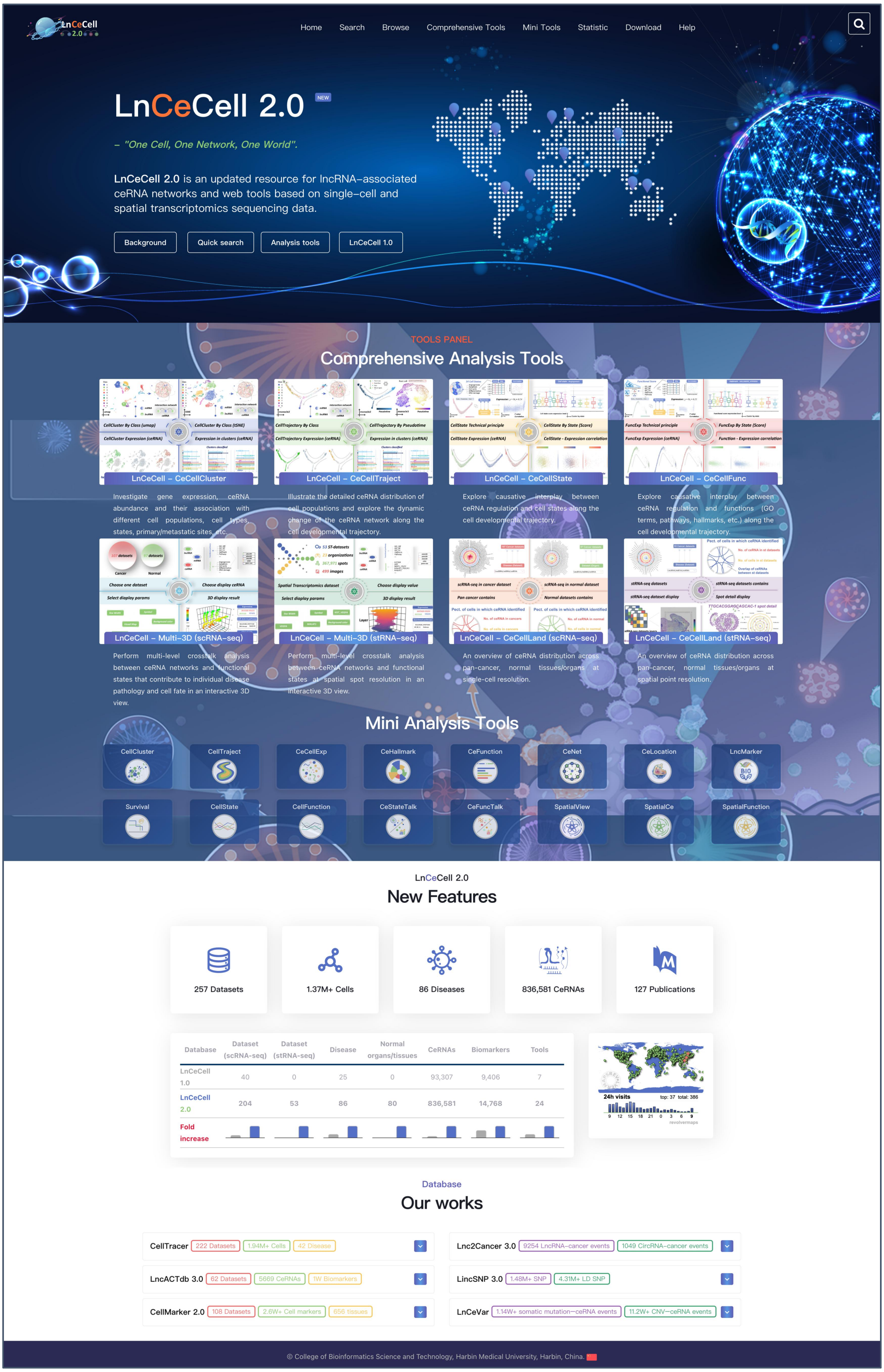

Figure S6. A screenshot highlighting the new features on the updated 'HOME' page of LnCeCell 2.0.

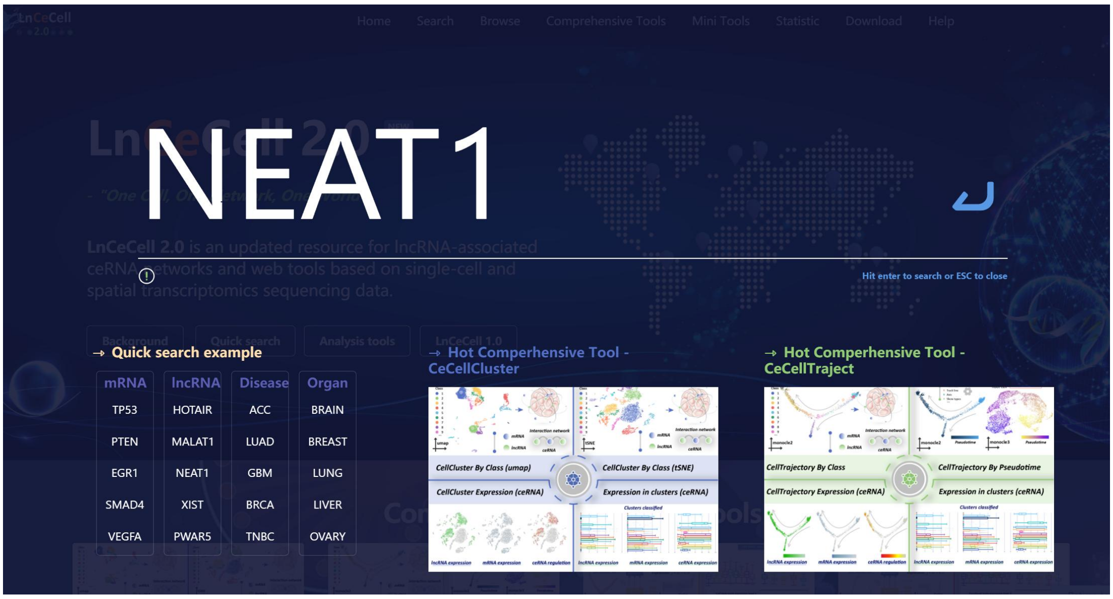

Figure S7. A screenshot of the 'QUICK SEARCH' page in LnCeCell 2.0, using the lncRNA NEAT1 as an example.

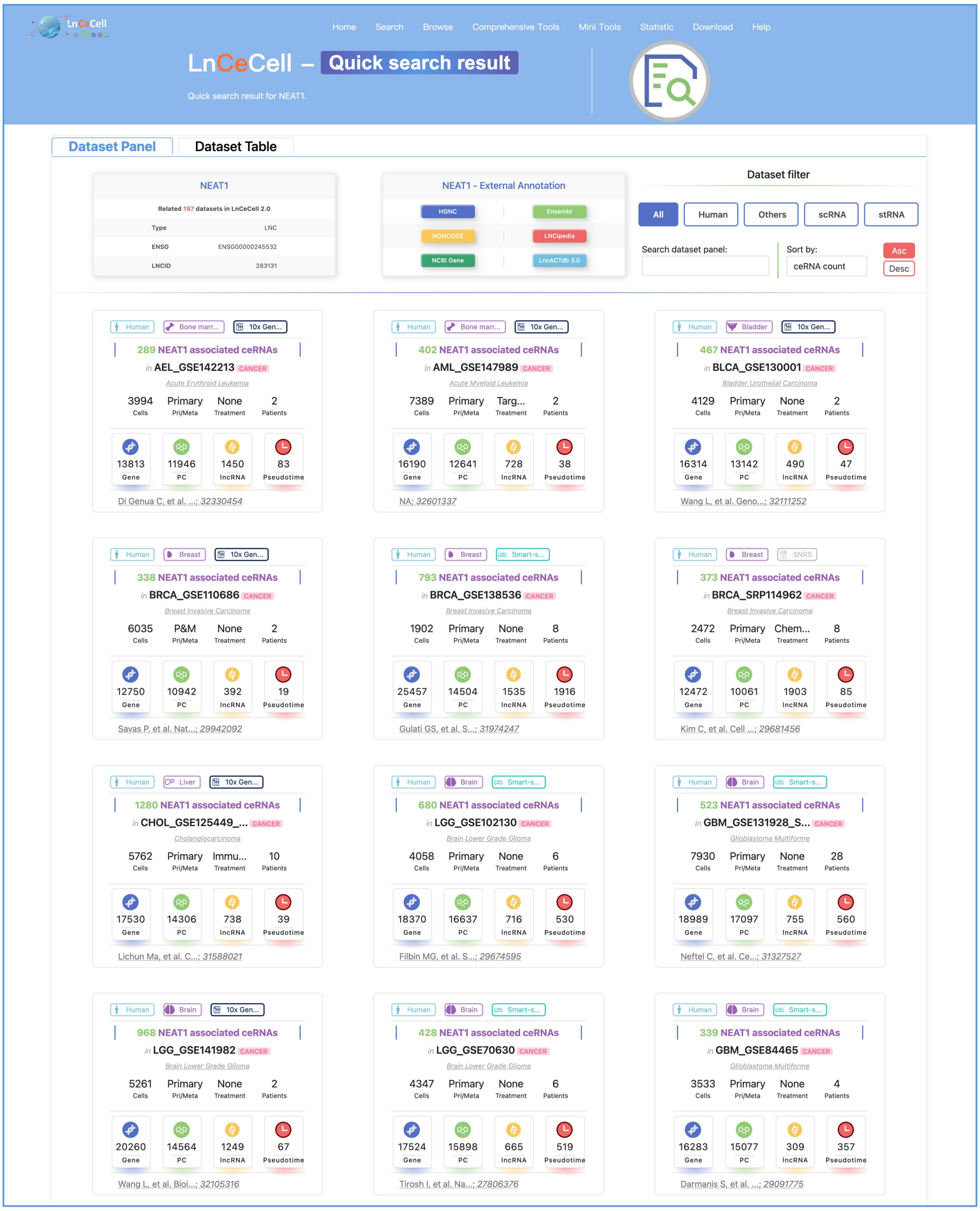

Figure S8. Data panels related to NEAT1 as illustrated by LnCeCell 2.0.

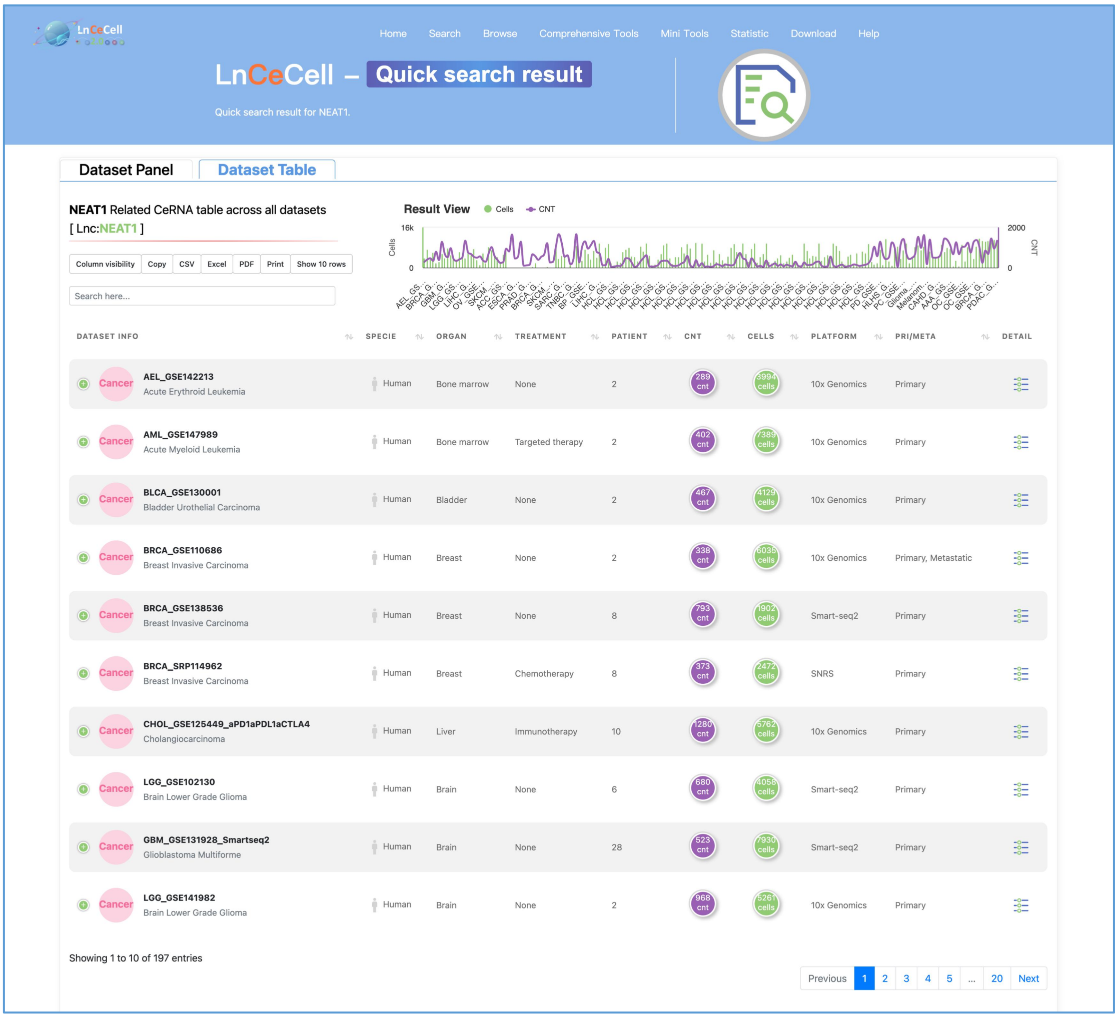

Figure S9. A data table listing information related to NEAT1 as provided by LnCeCell 2.0.

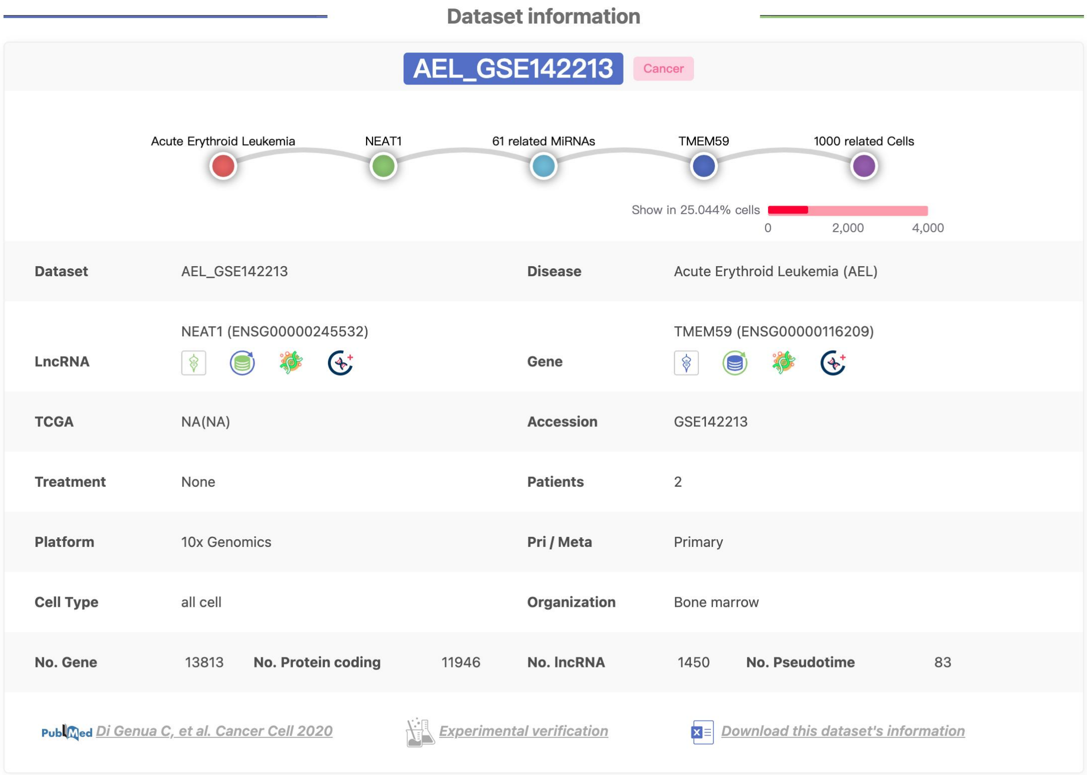

Figure S10. The detailed page in LnCeCell 2.0, containing comprehensive information on disease-ceRNA associations, diagnostic and treatment procedures, experimental-supported annotations, and the number and percentage of cells.

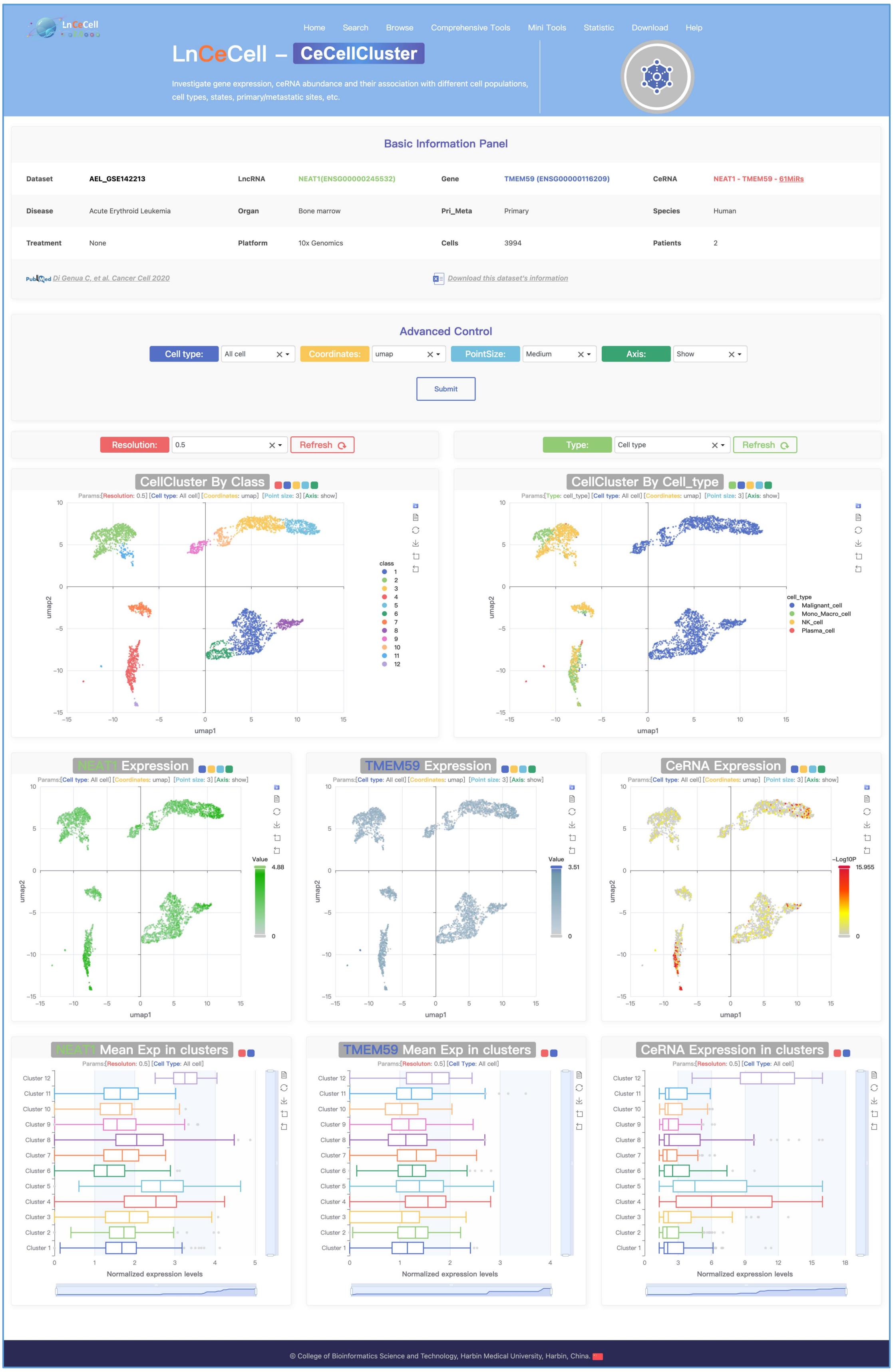

Figure S11. Comprehensive mapping of NEAT1 expression and its associated ceRNA interactions in diverse cell populations, illustrated by the *CeCellCluster* tool.

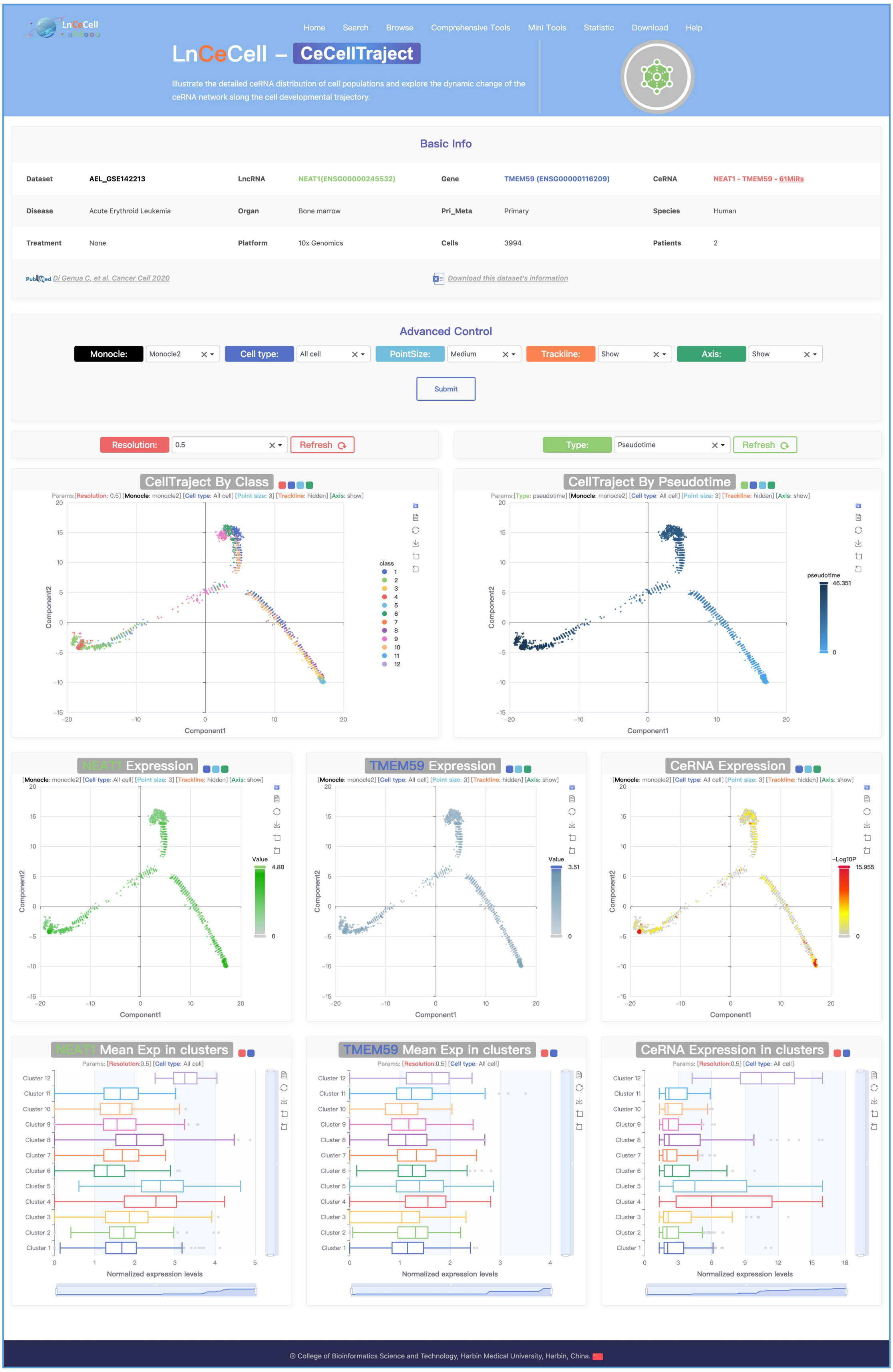

Figure S12. The dynamics of NEAT1-related ceRNA networks and their associations within different cell state lineages, constructed and illustrated using the *CeCellTraject* tool.

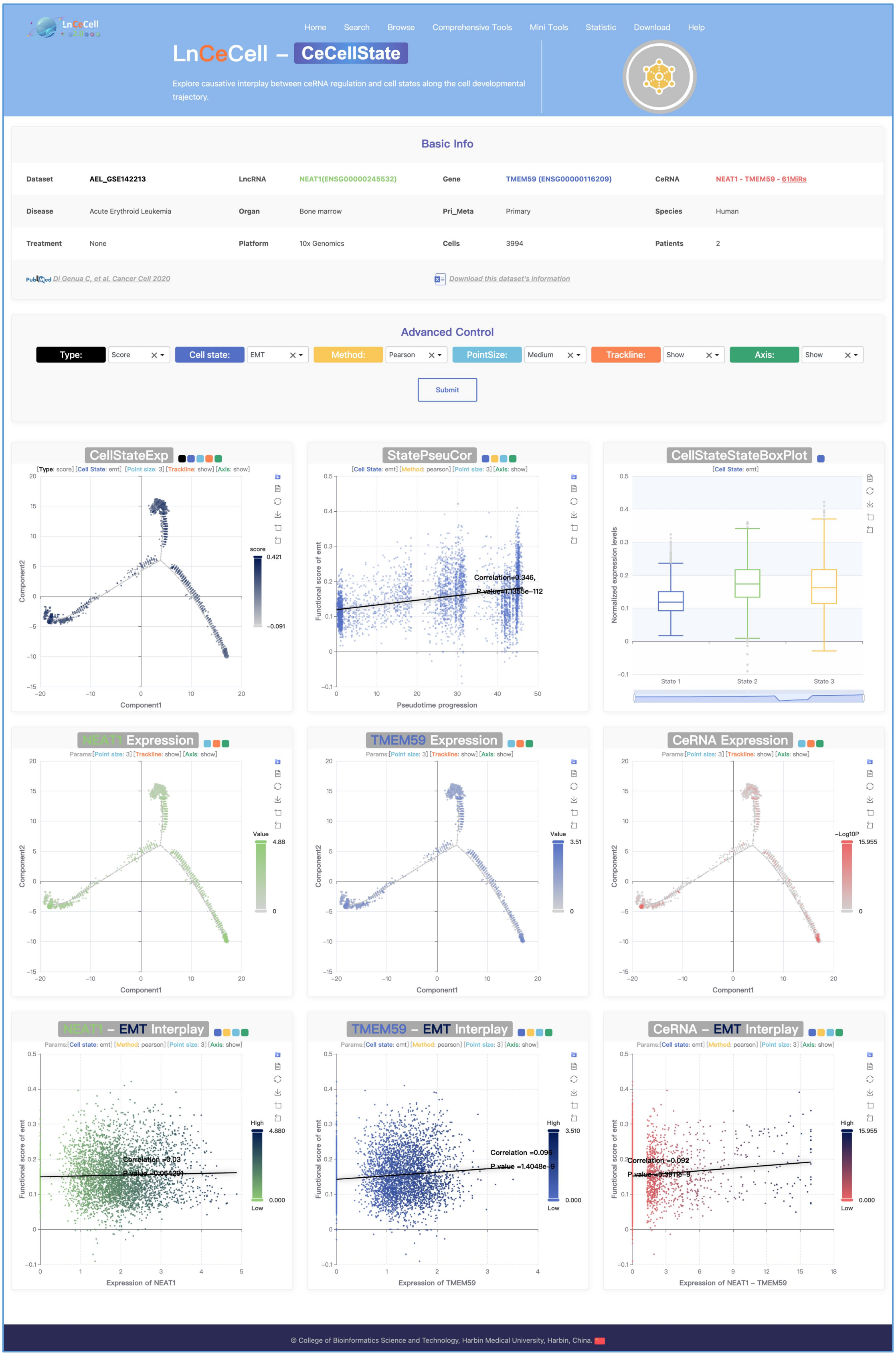

Figure S13. Identification of a positive correlation between NEAT1 expression and EMT score using the *CeCellState* tool.

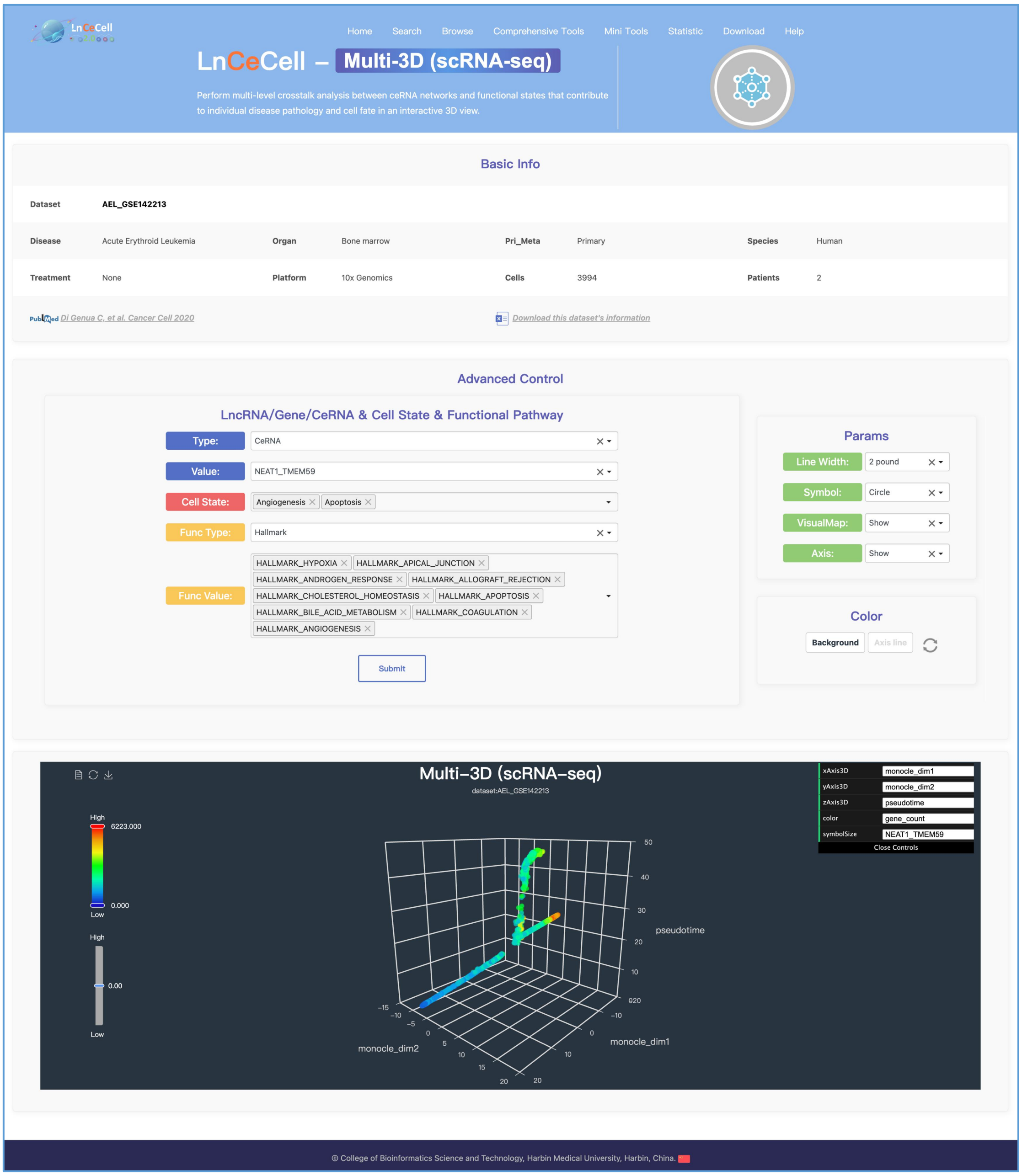

Figure S14. The Multi-3D (scRNA-seq & stRNA-seq) tool enables examination of the complex interplay between NEAT1-associated ceRNA networks and functions (such as GO terms, biological pathways, cancer hallmarks) that influence individual disease pathology and cell fate.

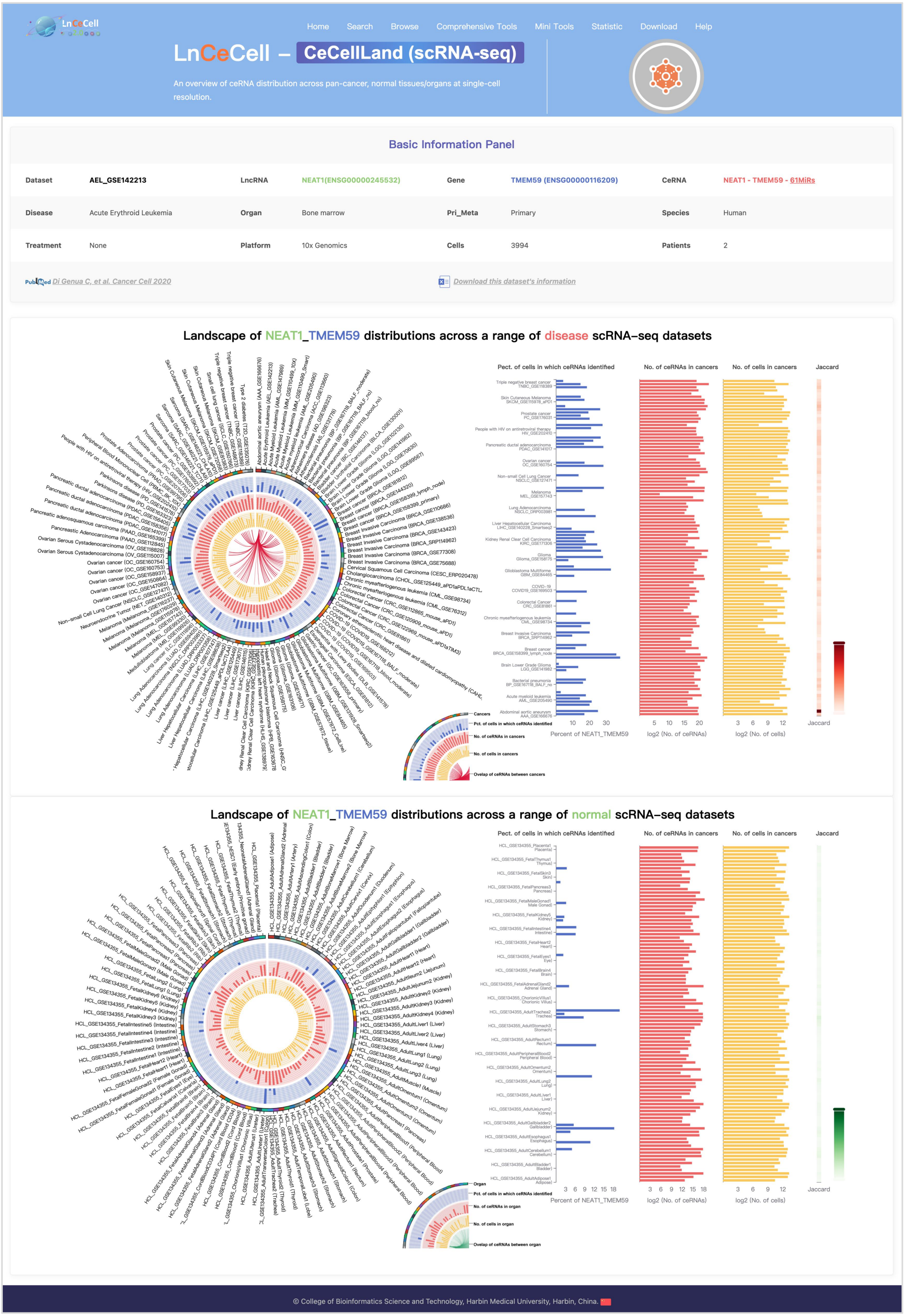

Figure S15. The CeCellLand (scRNA-seq & stRNA-seq) tool provides a global view of NEAT1-related ceRNA networks across a range of pan-cancers and normal tissues/organs.
